# Noninvasive sub-organ ultrasound stimulation for targeted neuromodulation

**DOI:** 10.1038/s41467-019-08750-9

**Published:** 2019-03-12

**Authors:** Victoria Cotero, Ying Fan, Tea Tsaava, Adam M. Kressel, Ileana Hancu, Paul Fitzgerald, Kirk Wallace, Sireesha Kaanumalle, John Graf, Wayne Rigby, Tzu-Jen Kao, Jeanette Roberts, Chitresh Bhushan, Suresh Joel, Thomas R. Coleman, Stavros Zanos, Kevin J. Tracey, Jeffrey Ashe, Sangeeta S. Chavan, Christopher Puleo

**Affiliations:** 1 GE Global Research Center, 1 Research Circle, Niskayuna, NY 12309 USA; 2 Feinstein Institute for Medical Research, 350 Community Drive, Manhasset, NY 11020 USA

**Keywords:** Ultrasound, Peripheral nervous system, Inflammation

## Abstract

Tools for noninvasively modulating neural signaling in peripheral organs will advance the study of nerves and their effect on homeostasis and disease. Herein, we demonstrate a noninvasive method to modulate specific signaling pathways within organs using ultrasound (U/S). U/S is first applied to spleen to modulate the cholinergic anti-inflammatory pathway (CAP), and US stimulation is shown to reduce cytokine response to endotoxin to the same levels as implant-based vagus nerve stimulation (VNS). Next, hepatic U/S stimulation is shown to modulate pathways that regulate blood glucose and is as effective as VNS in suppressing the hyperglycemic effect of endotoxin exposure. This response to hepatic U/S is only found when targeting specific sub-organ locations known to contain glucose sensory neurons, and both molecular (i.e. neurotransmitter concentration and cFOS expression) and neuroimaging results indicate US induced signaling to metabolism-related hypothalamic sub-nuclei. These data demonstrate that U/S stimulation within organs provides a new method for site-selective neuromodulation to regulate specific physiological functions.

## Abstract (web summary)

Stimulation of peripheral nerve activity may be used to treat metabolic and inflammatory disorders, but current approaches need implanted devices. Here, the authors present a non-invasive approach, and show that ultrasound-mediated stimulation can be targeted to specific sub-organ locations in preclinical models and alter the response of metabolic and inflammatory neural pathways.

## Introduction

**Fig. 1:**
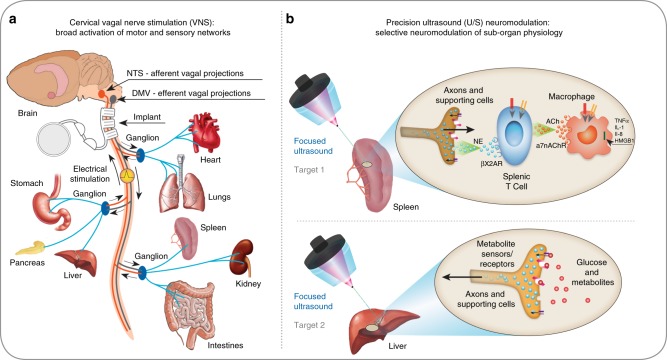
Implant-based vagus nerve stimulation (VNS) versus precision ultrasound (U/S) neuromodulation. **a** A schematic of the neurons within the vagus nerve, exemplary innervated organs, and the common cervical position used for VNS devices. Stimulation of the cervical vagus results in stimulation of both target and non-target efferent and afferent pathways^1–10^. Clinical implementation of miniature stimulators and advanced electrode designs that can be implanted closer to the target organ (for precision stimulation of axons entering only that organ) is challenging and remains elusive^9,17,18^. **b** A schematic of precision organ-based neuromodulation in which the innervation points of known axonal populations are targeted for stimulation using focused pulsed U/S. Targets investigated herein include innervation points within the spleen and sensory terminals within the liver

Every organ contains nerves that regulate the organ’s function. New medical devices are under development that modulate signals on such nerves to treat disease^1,2^ (e.g., inflammatory^3^, hypertension^4^, diabetes^5^, obesity^6^, and gastrointestinal disorders^7,8^). However, nerve stimulation strategies using permanently implanted electrodes^3,9,10^, transcutaneous electro-magnetic fields^11,12^, or adapted brain stimulation technologies^13–16^ are limited to stimulating large nerves that can be accessed by an implanted device (Fig. 1a) or nerves close to the surface of the skin.

The anatomical structure of the peripheral nervous system (PNS) presents difficult challenges. Within peripheral nerves, individual axons are tightly bundled in groups (fascicles) and wrapped within protective tissue. This makes it difficult to selectively stimulate subsets of axons that terminate in specific organs and uniquely modulate the function of communicating cells within that organ. Researchers have attempted to develop increasingly advanced electrode designs^9,17^ or miniature stimulators^18^ to implant on smaller nerves near the target. However, clinical implementation of precision peripheral nerve stimulation remains elusive. For example, studies of vagus nerve stimulation (VNS) depend on cervical implants (Fig. 1a) that activate a broad mixture of efferent^1,3,5–7,19–22^ and/or afferent^5,6,21–23^ neural pathways. And, despite promising VNS trials, low-precision stimulation protocols have not allowed the direct association of clinical observations with specific nerve targets without non-specific stimulation of other (non-target) neural pathways^3,6,21,22^. New nerve-stimulation methods are needed to non-invasively stimulate specific targets and correlate organ-specific neural activity with function for broad clinical translation.

Previous attempts to utilize U/S to directly stimulate peripheral nerves alone have focused U/S energy on the same large nerves (outside the organ) that host implanted electrodes, and this strategy has produced conflicting results. Ultrasound focused on ex vivo nerves has been shown to stimulate nerves (i.e., elicit action potentials) under a limited set of conditions^23–25^, or at powers that may cause damage to the nerve and/or surrounding tissue^24,26^. At the same time, in vivo studies have either failed to elicit nerve activation^23^, or used indirect methods of measuring nerve activation via neuromuscular pathways (i.e., measures of U/S-induced EMG signals or muscle movement)^27,28^. In contrast, several reports have demonstrated successful activation of end axons or nerve terminals in brain tissue^29^ and retina^30^. And, recently the Okusa group has shown activation of the cholinergic anti-inflammatory pathway (CAP)^1,3,6,10^ using a splenic ultrasound imaging protocol; successfully preventing renal ischemia-reperfusion injury in a mouse model of acute kidney injury (AKI) using an ultrasound imaging scan^31^. The group linked the ultrasound effect back to CAP activation through a series of control experiments, including splenectomy, adoptive transfer studies of CD4(+) T cells, α7 nicotinic acetylcholine receptor blockade, and genetic knockout^31^.

Herein, U/S energy is focused directly on specific anatomical targets of neural innervation (Fig. 1b) within the spleen and liver (without scanning the ultrasound transducer). As discussed above, neural innervation within the spleen is thought to affect systemic inflammation through the CAP^6,19,20^. Nerves in the liver are thought to communicate to the brain and provide a critical component of the nutrient sensing within the glucoregulation system^32^. Herein, we demonstrate that both pathways can be modulated with targeted U/S, and that the non-invasive U/S technique alleviates endotoxin-induced cytokine production and hyperglycemia at levels commensurate with traditional, invasive VNS. Furthermore, unlike cervical VNS (which broadly stimulates multiple vagal pathways), precision ultrasound neuromodulation enables separate modulation of the splenic (anti-inflammatory) versus the hepatic (metabolic) pathways. The splenic stimulation data demonstrates a clear ultrasound “dose response” to norepinephrine with a distinct power level required for cytokine reduction in the endotoxin model, and an effective power range commensurate with potential clinical use. Both the splenic and hepatic stimulation data are presented with direct measurements of U/S-induced neuromodulation (i.e., neurotransmitter concentration in the spleen experiments, and cFOS and DfMRI data in the liver experiments), and indirect measurements of effects on down-stream signaling pathways (i.e., kinase activities for several important/relevant intracellular signaling pathways). Finally, chemical/mechanical blocking and genetic knock-out experiments are shown for several signaling components in the splenic pathway, and data on the effect of these knock-outs on ultrasound-induced activation of CAP is reported. These results, combined with our companion paper by Zachs et al. (that demonstrates the use of splenic U/S stimulation to reduce disease severity in a preclinical model of inflammatory arthritis), provide the most thorough report to date on the potential for precision ultrasound stimulation to replace implantable devices for the translation of peripheral neuromodulation-based therapies^1,2^.

## Results

### Targeted sub-organ ultrasound neuromodulation

**Fig. 2:**
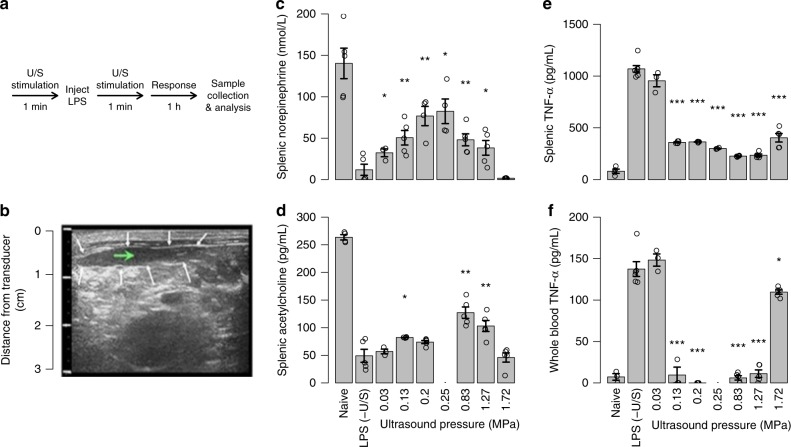
Splenic U/S neuromodulation of the cholinergic anti-inflammatory pathway (CAP). **a** The timeline of the U/S neuromodulation performed in the LPS-induced inflammation model (see Methods and Supplementary Figures 1–6 for details; stimulation parameters were 1.1 MHz, 136.36 µs pulse length, and 0.5 ms pulse repetition period). **b** Example U/S image of the spleen used to locate the U/S stimulus (white arrows—outline of the spleen; green arrow—target point for U/S stimulation). **c**–**e** Splenic concentrations of CAP signaling molecules, including norepinephrine (**c**), acetylcholine (**d**), and TNF (**e**) are shown for naive animals, sham controls (LPS, -U/S), and with U/S stimulation (0.03–1.72 MPa). **f** Whole-blood concentrations of TNF for the same conditions as (**e**). The asterisks mark statistical significance using two-sided *t*-test versus LPS only controls (with *p*-value thresholds; *p < 0.05, **p < 0.01, ***p < 0.001). *n* = 5 for all experiments in this figure except for all LPS − (U/S) controls which were *n* = 7

In splenic stimulation experiments, the output of each cell type involved in the CAP (using the LPS-induced acute inflammation rodent model under different U/S stimulation conditions) was monitored (Fig. 2a, b; see Methods for details of U/S stimulation and the LPS model^6,19–22^). The CAP (Fig. 1b) includes three major cell types: the end axon terminals that project from the splenic ganglia, intermediary T-cells, and macrophages that release cytokines systemically. Electrical stimulation of this pathway is well studied; neuromodulation affects cytokine production and potentially provides therapeutic benefit in several chronic inflammatory diseases^3,7^. Figure 2b shows an example U/S image used to non-invasively target the U/S stimulus (see Methods and Supplementary Figures 1–5 for details on image guidance). Unlike previous examples of ultrasound stimulation^31^, the ultrasound stimulus was not swept across the organ or focused on a large nerve outside the organ, but rather targeted to specific locations of nerve innervation within the organ (see Supplementary Figure 5 for details of estimated stimulation areas within the organs). We evaluated CAP response to the targeted US stimulation by measuring splenic concentrations of CAP-related neurotransmitters and cytokines including norepinephrine (NE), acetylcholine (ACh), and tumor necrosis factor (TNF; see Methods for ELISA and HPLC procedures; see Supplementary Figure 6 for data in LPS-naive animals). A 1.1 MHz U/S transducer was focused directly on splenic targets using U/S coupling gel. Figure 2c–f shows the measured CAP response in control rodents (those having received LPS and not U/S stimulation), and in U/S-treated rodents. Splenic NE levels averaged 140 nmol/L in naive animals, whereas the LPS controls dropped NE levels to near zero, demonstrating suppression of CAP signaling during LPS-induced inflammation. The U/S stimulus attenuated the LPS response toward levels measured in naive animals (Fig. 2c). Consistent with the CAP signaling process, the NE increase in the U/S-stimulated animals correlated with a splenic ACh increase and, at 0.83 MPa U/S pressure, the average ACh concentration was nearly three times that found in the sham animals (Fig. 2d). Also, both splenic (Fig. 2e) and circulating (Fig. 2f) TNF levels were significantly reduced compared to the sham animals. Treatment response depended on U/S pressure, and the dose-response curve showed the most effective pressure parameters to range between 0.25 and 0.83 MPa (parameters corroborated in our companion paper by Zachs et al.).

**Fig. 3:**
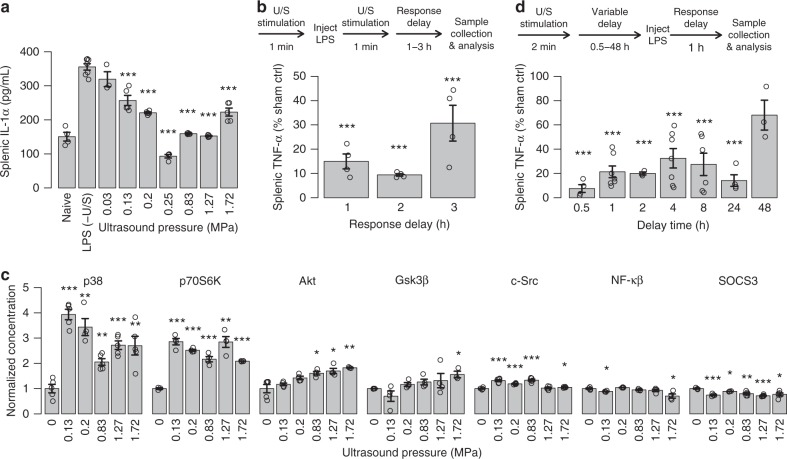
Lasting effect of splenic U/S neuromodulation. **a** Splenic IL-1α concentrations measured from the same samples as Fig. 2c–e. *n* = 5 for all experimental conditions, except LPS–(U/S) controls which were *n* = 7. **b** Study timeline and data designed to measure the concentrations of splenic TNF after response times of 1–3 h (i.e., the time the sample was harvested post treatment). *n* = 4 for each experimental condition. **c** Normalized concentrations (compared to LPS controls) of activated/phosphorylated kinases with or without U/S stimulus pressures from 0.03 to 1.72 MPa. *n* = 4 for each experimental condition. **d** Concentration of splenic TNF after protective (pre-LPS) U/S treatments after delay times from 0.5 to 48 h prior to LPS injection. *n* = 5. The asterisks mark statistical significance using two-sided *t*-test versus LPS only controls (with *p*-value thresholds; *p < 0.05, **p < 0.01, ***p < 0.001). All experiments in this figure were performed using the same US settings as in Fig. 2 (0.83 MPa)

The broader impact of U/S stimulation on CAP-related intra- and extracellular signaling pathways, and the kinetics of the U/S effect were also investigated. In addition to TNF, U/S stimulation also significantly reduced splenic interleukin-1-alpha (IL-1α) levels (Fig. 3a). The maximum U/S-mediated response to LPS-exposure occurred 1–2 h after treatment (Fig. 3b), showing kinetics similar to previous implant-based-VNS studies^6,19–21^. To further characterize the effect, we measured U/S activation of specific intracellular kinases (Fig. 3c) that are associated with LPS-^33^, CAP-^34^, or TNF-α-mediated^35^ signaling. These data show that U/S strongly enhanced activation of some kinases (e.g., p38 and p70S6K), and the U/S pressure-dependent response of some kinases (e.g., p38) approximately correlated with the U/S pressure dependence of neurotransmitter and cytokine concentrations previously observed (Fig. 2c–e). Finally, splenic U/S showed a persistent suppressive effect when applied before the LPS injection (Fig. 3d), also consistent with previous invasive VNS studies^6,19–21^. Figure 3d shows that this protective effect continued well after treatment, and a single U/S treatment produced a statistically reduced TNF response to endotoxin up to 48 h after U/S stimulation. In corroboration, our companion paper by Zachs et al. (using a serum-transfer arthritic mouse model), independently showed that non-invasive splenic U/S stimulation alleviated the severity of arthritis, and that this ultrasound-specific effect (using the same optimal ultrasound frequency, and similar stimulation pressure) was apparent when administered before or after induction and manifestation of the disease.

**Fig. 4:**
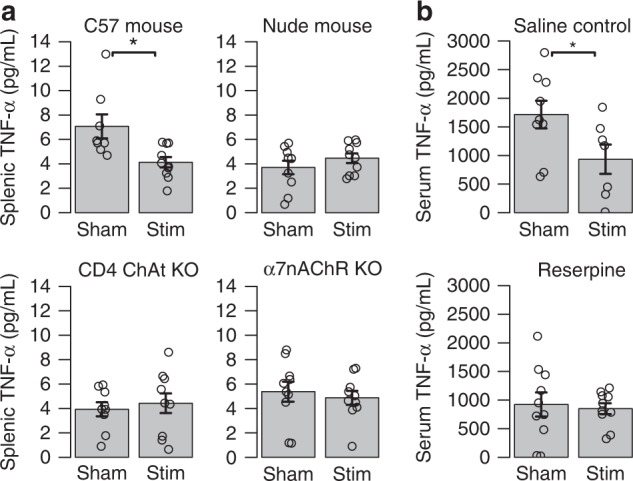
Splenic U/S stimulation suppresses systemic TNF levels during endotoxemia through CAP. **a** Splenic concentrations of TNF are shown for sham controls (LPS, -U/S) and U/S stimulated mice (0.83 MPa ultrasound setting) for C57black/6 mice, Nude mice, *CD4 ChAT* knock-out mice, and α7nAChR knock-out mice. **b** Serum TNF concentrations are shown for sham controls (LPS, -U/S) and U/S stimulated mice (0.83 MPa) ultrasound setting for saline injection controls and reserpine treated/denervated (see Methods for details) mice. All experiments in this figure were performed using the same U/S settings as in Figs. 2 and 3 (0.83 MPa)

To further confirm the role of the CAP in mediating the suppressive effects of splenic U/S stimulation, we performed tests in additional knock-out and denervation models (Fig. 4), including nude mice (lack functional T cells; Fig. 4a), *CD4 ChAT* (choline acetyltransferase) knock-out mice (genetic ablation of C*h*At in CD4+ T cells Fig. 4a), α7nACh receptor (Fig. 4a) knock-out mice, and reserpine-treated mice (inhibited catecholamine production; Fig. 4b). The Methods section contains descriptions of the methods used to create each knock-out model, and the protocol for reserpine-based depletion of catecholamine stores. Splenic U/S stimulation induced significant suppression of endotoxin-induced systemic TNF levels in wild type C57black/6 mice (Fig. 4a), similar to that observed in the endotoxemic rats (Fig. 2). To study the role of T cells, we carried out splenic U/S stimulation in nude mice. Splenic U/S stimulation failed to induce suppression of LPS-induced TNF in nude mice indicating that functional T cells are required for U/S-mediated suppression (Fig. 4a). Next, to identify whether acetylcholine-producing T cells are required for U/S-mediated suppression, we used *Cre-loxP* recombination in mice to selectively ablate C*h*At in CD4+ T cells. Splenic U/S stimulation in *CD4 ChAT* KO mice did not alter TNF levels significantly, as compared with controls indicating that U/S stimulation requires acetylcholine producing T cells for mediating TNF suppression (Fig. 4a). Macrophages are a major source of TNF production during endotoxemia, and *α7nAChR* expressed on macrophages plays a critical role in mediating cholinergic anti-inflammatory signaling^36^. Accordingly, we tested the effect of splenic U/S stimulation in animals lacking *α7nAChR* expression. As shown in Fig. 4a, U/S stimulation did not suppress TNF levels during endotoxemia in α7nAChR KO mice. Splenic U/S stimulation leads to a significant increase in splenic norepinephrine levels in endotoxemia model (Fig. 2b). To further assess the role of catecholamines in this pathway, we performed splenic U/S stimulation in endotoxemic mice previously depleted of catecholamine stores by treatment with reserpine. Splenic U/S stimulation attenuated systemic TNF levels in control animals but not in reserpine-treated animals (Fig. 4b). Together, these findings indicate that splenic U/S stimulation attenuates TNF production during endotoxemia through CAP and provide further evidence of a neuromodulatory mechanism versus a direct ultrasound effect on splenic immune cells.

**Fig. 5:**
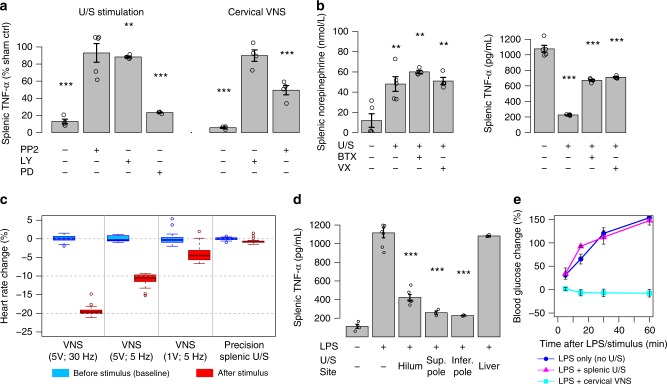
Comparison of splenic U/S stimulation versus traditional cervical VNS of CAP. **a** Relative concentrations of splenic TNF are shown for US-stimulated (left; stimulation at 0.83 MPa) versus implant-based VNS (see Methods for details) treated animals (concentrations are shown as a percent change relative to LPS-treated sham stimulation controls). The first bar on the left of each graph shows the effect of ultrasound versus VNS on attenuation of LPS-induced inflammation without the addition of any blockers or inhibitors. The remaining bars show the effect of pre-injection of the inhibitors PP2 (4-amino-5-(4-chlorophenyl)-7-(dimethylethyl)pyrazolo[3,4-d]pyrimidine, partially selective for Src kinase), LY294002 (PI3-kinase selective), and PD98059 (MEK1- and MEK2-selective MAPK inhibitor). *n* = 4 for each experimental condition. **b** Data showing the effect of α-bungarotoxin (BTX) or surgical (cervical) vagotomy on splenic concentrations of (left) norepinephrine (NE) and (right) TNF after US stimulation. *n* = 4 for each experimental condition. **c** Data comparing the effect of VNS (at several intensities and frequencies) versus splenic US stimulation (at 0.83 MPa) on heart rate (see Methods). **d** Data showing specificity in the modulated TNF response for splenic versus liver U/S stimulation, but similar TNF response shown at different splenic stimulation locations (i.e., splenic hilum, superior and inferior pole). *n* = 3 for each experimental condition. **e** Data confirming the VNS side-effect on attenuation of LPS-induced hyperglycemia and absence of this side-effect when using splenic U/S stimulation. Relative blood glucose concentrations are shown compared to pre-injection concentration at times of 5, 15, 30, and 60 min for the unstimulated controls (blue-circles), splenic U/S stimulation (purple-triangles), or cervical VNS (light blue-squares). *n* = 12 for each experimental condition. All experiments in this figure were performed using the same U/S parameters as Figs. 3 and 4

The effect of splenic U/S neuromodulation was then compared to standard implant-based VNS (see Methods for VNS details). Figure 5a shows that invasive cervical VNS and noninvasive splenic U/S stimulation have a nearly equivalent effect on TNF (see the U/S stimulation (left) and VNS implant stimulation (right) bars without the addition of kinase inhibitors (- PP2, -LY, and -PD)). Furthermore, Fig. 5b shows that splenic injection of α-bungarotoxin (BTX, a known antagonist for the α7nACh receptor central to CAP signaling^19–22^) suppressed the effect of U/S stimulation on TNF-α concentration (demonstrating that, like VNS-based CAP activation^3,19–22^, optimal CAP modulation by U/S requires splenic α7nAChR signaling). Consistent with the CAP model (Fig. 1b), NE concentration was unaffected by BTX (i.e., BTX blocked the effect of elevated NE through the α7nAChR pathway, and not through modification of neurotransmitter release itself). Vagotomy also suppressed CAP modulation by U/S (Fig. 5b), providing suppression of the effect of U/S stimulation on TNF concentration to the same level as BTX. However, vagotomy failed to inhibit the effect of U/S on NE concentrations within the spleen during the LPS experiments, suggesting that cervical vagotomy may not immediately affect the ability of local splenic tissue (down-stream of the vagotomy) to respond to the ultrasound stimulus. This data showing attenuation of the U/S-induced TNF suppression without a change in post-U/S NE concentrations may also be explained by more recent studies of the vagal anti-inflammatory pathway suggesting other locations of the critical α7nACh receptor^22^. Finally, the kinase inhibitors PP2 (4-amino-5-(4-chlorophenyl)-7-(dimethylethyl)pyrazolo[3,4-d]pyrimidine, partially selective for Src kinase) and LY294002 (PI3-kinase selective) were shown to suppress the U/S effect, while PD98059 (MEK1- and MEK2-selective MAPK inhibitor) showed no effect (Fig. 4a). These results corroborate those in Fig. 3c, in which U/S stimulation altered kinase activation within the CAP^34^ and TNF^35^ related PI3 (i.e., Akt, P70S6K), c-Src, and p38-MAPK pathways, but not kinases involved in direct bacterial antigen response (i.e., GSK3B).

The physiological specificity of focused U/S stimulation was then investigated by measuring several known side effects of invasive VNS. Figure 5c shows the change in heart rate caused by cervical VNS or splenic U/S neuromodulation (see Methods for heart rate measurement details). At 2- and 5-V VNS intensities, heart rate significantly decreased. However, local splenic U/S neuromodulation showed no effect on heart rate. Stimulation of off-target sites (i.e., liver) did not modulate the LPS-induced TNF response; however, U/S stimulation at several different sites within the spleen provided similar modulation of the TNF response (Fig. 5d). Interestingly, Fig. 5e shows that splenic U/S stimulation had no effect on blood glucose concentration, whereas VNS experiments exhibited the previously-observed side effect of attenuating LPS-induced hyperglycemia. It is hypothesized that this metabolic side effect of CAP-targeted VNS^6^ may be due to off-target VNS of a second (non-CAP) vagal pathway.

### Precise stimulation of sensory sites in the liver

**Fig. 6:**
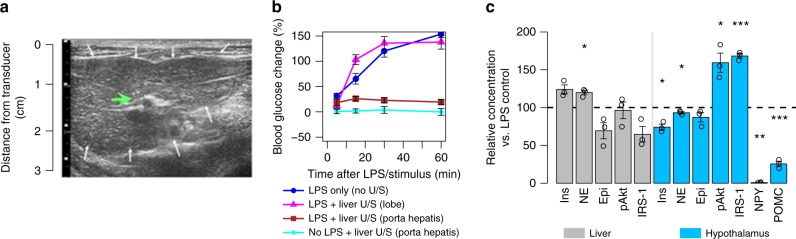
Hepatic U/S stimulation of pathways that affect glucose regulation. **a** 2D US image of the liver used to focus the U/S stimulus to the target site (green arrow; white arrows—outline of the liver). **b** Data showing the effect of U/S stimulation of the liver on LPS-induced hyperglycemia. Relative blood glucose concentrations compared to pre-injection concentration are shown at times of 5, 15, 30, and 60 min. The data show reversal of LPS-induced hyperglycemia after U/S stimulation of the porta hepatis (red-squares), but not distal lobes (purple-triangles), compared to LPS alone (blue-circles) or naive/no-LPS stimulated (light blue-diamonds) samples **c** Relative concentrations compared to no U/S for molecules associated with metabolism in the liver and hypothalamus. Significant response to U/S stimulation was found for norepinephrine (NE), protein kinase B (pAkt), insulin receptor substrate 1 (IRS-1), and neuropeptide Y (NPY) in the hypothalamus. The asterisks mark statistical significance using two-sided *t*-test versus LPS only controls (with *p*-value thresholds; *p < 0.05, **p < 0.01, ***p < 0.001). *n* = 12 for each experimental condition. All experiments in this figure were performed using the same U/S parameters as Figs. 3–5

VNS has been shown to reduce hyperglycemia^5,6^. To investigate whether this was associated with neuromodulation of hepatic sites, VNS was compared with our precision U/S stimulation technique applied to the liver. Figure 6a shows the U/S-image guidance that enabled locating the U/S stimulus at the porta hepatis region of the liver, which contains glucose-sensitive neurons known to signal to and modulate metabolic control centers within the hypothalamus^37^. We found that hepatic U/S stimulation provided protection against LPS-induced hyperglycemia, as Fig. 6b shows that U/S stimulation limited the increase in blood glucose levels to within post-prandial concentrations. Furthermore, this effect was anatomically specific. Figure 6b shows that locating the stimulus toward the right or left lobe of the liver reduced the effect of U/S stimulation. Furthermore, hepatic concentrations of local signaling molecules associated with glucose metabolism showed that U/S stimulation did not show direct changes within the liver indicative of direct modulation of hepatic glycolytic or glycogenolytic processes (Fig. 6c, gray bars). Instead, it was found that U/S stimulation of the sensory neuron containing porta hepatis region resulted in significantly reduced hypothalamic concentrations of NPY and increased hypothalamic insulin receptor substrate (IRS-1) and protein kinase B (pAkt) activation (Fig. 6c, blue bars). The increase in IRS and pAkt phosphorylation indicates increased insulin signaling in the hypothalamus, which is capable of driving the observed reduction in concentrations of NPY. These results are consistent with previous reports of LPS-induced dysregulation of insulin-mediated IRS1-P13 signaling^38^. Interestingly, these results also suggest that altered signaling through the central nervous system may be involved in the role that chronic inflammation (i.e., metabolic endotoxemia) has been shown to play in the pathogenesis of insulin resistance^39,40^.

**Fig. 7:**
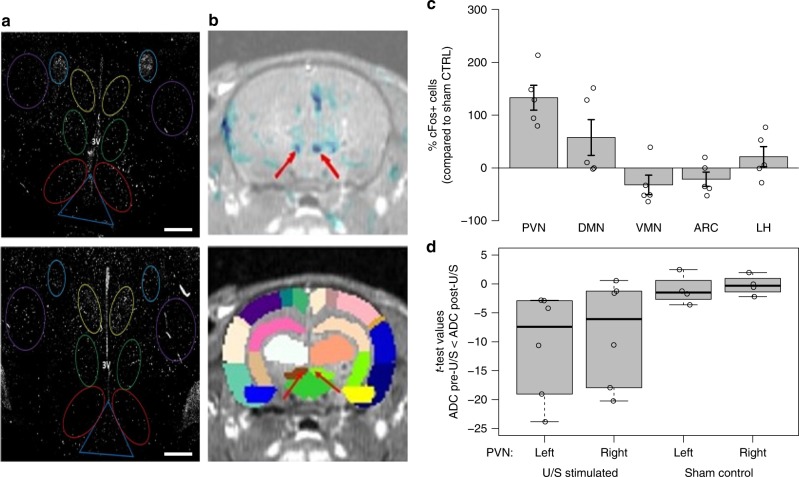
Histochemical and DfMRI analysis of neural pathways associated with response to hepatic U/S stimulation. **a** cFOS immunohistochemistry images showing the number of activated neurons in the (top) unstimulated control and (bottom) U/S-stimulated animals. Images were segmented on the paraventricular nucleus (yellow; PVN), dorsal medial nucleus (green; DMN), ventromedial nucleus (red; VMN), arcuate nucleus (dark blue; ARC), and lateral hypothalamus (purple; LH). Scale bar = 300 microns. **b** Example MRI overlays between activation maps and the T1 SPGR volume (top; see Methods for details) and a brain atlas overlay on the T1 SPGR volume (bottom; see Methods, Supplementary Figures 13 and 14, and Supplementary Table 2 for further details). The blue color in the top image denotes regions where ADC changed significantly post-U/S. Each color in the bottom image represent anatomically distinct brain regions in the atlas; major areas showing decreased ADC (top image; red arrows) aligned with the left (brown) and right (light green) PVN in the bottom image (red arrows). **c** Data showing the percent change of the number of cFos expressing cells in the US stimulated animals (*n* = 8) compared to sham controls (*n* = 6) in each of the segmented hypothalamic regions (PVN, DMN, VMN, ARC, and LH), images in (**a**) represent one set of sham versus stimulated paired animals. **d** *T*-test values from corresponding-pixel comparison within PVN ROIs (see Methods for details) between the pre- and post-treatment ADC maps showing increased ADC values (compared to controls) for the 6 animals tested after U/S stimulation, images from (**b**) represent results from one exemplar animal. All experiments in this figure were performed using the same U/S parameters as Figs. 3–6, except the DfMRI experiment in which a MR compatible ultrasound transducer was needed. The compatible ultrasound transducer had an acoustic frequency of 1.47 MHz. The band inside the box shows the second quartile, while the whiskers represent the minimum and maximum of all the data

Having observed this response in the brain, we sought to verify that neurons within afferent pathways were being modulated upon hepatic U/S stimulation (Supplementary Figure 11 contains a description of known afferent and efferent neural pathways involved with glucose regulation and homeostasis that interact with the hypothalamus). The U/S-induced neuromodulation was first quantified by measuring the expression of the immediate early gene c-Fos within defined hypothalamic and brainstem sub-nuclei known to modulate glucose homeostasis (Fig. 7a, c and Supplementary Figure 12, respectively)^37,41^. Compared to the controls, there was significant changes in the number of c-Fos positive (c-Fos+; Fig. 6a) cells within the paraventricular nucleus (PVN), dorsomedial nucleus (DMN), ventromedial nucleus (VMN), arcuate nucleus (ARC), and lateral hypothalamus (LH), suggesting U/S-induced modulation of the LPS-induced glucoregulatory neural signaling. These data corroborate the previous finding that ultrasound stimulation is modulating hypothalamic insulin sensitivity (Fig. 6c), which results in changes in nerve activity that are known to alter signals to the PVN (and outgoing or peripheral metabolic regulation)^37–39,42,43^. Furthermore, the altered hypothalamic c-Fos expression was accompanied by increased c-Fos expression within the nucleus tractus solitaris (NTS; Supplementary Figure 12), suggesting U/S-mediated modulation via signaling through afferent pathways (see Methods for immunohistochemistry details).

To further support the previous finding, the apparent diffusion coefficient (ADC) from diffusion-weighted functional magnetic resonance imaging (DfMRI) images in the hypothalamic sub-nuclei were compared before and after hepatic U/S stimulation. DfMRI^44–46^ is a technique that is still under development that has recently been shown sensitive to changes in nerve activity driven by external neuromodulators^47^ (see Methods, Supplementary Figures 13 and 14, and Supplementary Table 2 for DfMRI details). In response to the U/S stimulus, the ADC changed significantly within the PVN, corroborating both the chemical (POMC, NPY) and c-Fos expression data, which demonstrated U/S-induced modulation of LPS-activated pathways communicating to the hypothalamus. Figure 7b shows an exemplary *t*-test map (top image) showing this U/S-mediated change in ADC (suggesting changes in ultrasound modulated neural activity^44–47^). Figure 7d summarizes the ADC change within the PVN across all experiments (including data from the animal shown in Fig. 7b). DfMRI (though newly developed^47^) is useful herein in examining changes in nerve activity driven by the external ultrasound neuromodulation. And, when viewed together the DfMRI, cFos, and hypothalamic neurochemistry results, provide a set of data that are mutually consistent with ultrasound activation of a pathway that modulates the LPS-induced effect on energy metabolism via the NPY system and its effect on outgoing PVN signaling^42,43^.

## Discussion

In this work, we demonstrated noninvasive neuromodulation through sub-organ U/S stimulation of targeted sites of nerve innervation. Our technique uses focused US to target the innervation points and areas of specific synaptic connection and physiological function. This approach employs the natural hierarchical structure and organization of the nervous system, enabling precision neuromodulation with a noninvasive stimulation technology. Ultrasound modulation of two completely different organs and distinct physiological pathways (the CAP in the spleen and metabolic sensory neurons/cells in the liver) was achieved with the same technology. This opens the potential for applying this method to modulate other peripheral signaling pathways, providing a new U/S tool to map the structure–function relationship between the PNS and physiological organ function in health and disease.

With respect to the splenic CAP pathway, a recent study has shown that enhancement of CAP signaling through implant-based VNS inhibits production and circulation of cytokines (i.e., TNF, IL-1β, and IL-6) in humans^3^, as it did in the previous pre-clinical models^1,2,6,7,10,21,22^. In addition, VNS significantly inhibited TNF production in a whole blood assay for up to 84 days, and positively affected disease severity in a pilot study with rheumatoid arthritis patients^3^. As the first of its kind study, it shows the potential to use neuromodulation therapies in diseases that are currently treated with drugs. However, larger studies will be necessary to further analyze risk versus benefit for using implantable nerve stimulators to replace pharmacological treatments.

As an interesting alternative, researchers in our companion paper by Zachs et al. showed that splenic ultrasound stimulation was effective in reducing disease severity in a mouse model of inflammatory arthritis (using chosen U/S stimulation parameters and intensities that are substantiated by our data). A separate research group^31^, has also recently published the use of an imaging or transducer scanning method of applying ultrasound to the spleen to activate CAP and modify disease (i.e., prevention of renal ischemia-reperfusion injury in a mouse model of AKI). Herein, we demonstrated that precision ultrasound application (without scanning the transducer or ultrasound energy) is also adequate to activate CAP. In addition, results from the reserpine/catecholamine depletion experiments demonstrated that this effect is dependent on ultrasound interaction with the nervous system (or interfacing cells), providing further evidence of use of the technique as a non-invasive method of neuromodulation. This first report of precision or local nerve activation (using a tightly focused and image targeted ultrasound beam) demonstrates the potential to use ultrasound as a less invasive alternative to implant-based neuromodulation and opens novel paths toward clinical translation of bioelectronic medicines^1,2^.

To demonstrate this potential, our team applied the precision/image-targeted ultrasound stimulus to a non-CAP nerve target for the first time (the hepatic target, previously hypothesized to harbor glucose sensory neurons^37,41^). Our results corroborate previous studies that utilized glucose clamp methods to control concentrations and/or fluctuations in circulating glucose levels, and thus signaling from those glucose/nutrient sensing neurons in peripheral organs^37,43^. These early clamping techniques (which require isolation of local blood circulation, such as portal blood, from systemic blood) are difficult, and therefore limit the number of studies that attempt to identify the specific roles of peripheral sensory neurons in metabolic homeostasis. In contrast, the technique described herein represents a new method for modulating peripheral sensory output experimentally and may enable a new wave of research on the contribution of peripheral sensing and signaling in health and disease.

Continued work will be needed to further explore the potential of precision ultrasound neuromodulation. Supplementary Figure 5 provides schematics showing the level of precision obtained herein in stimulating specific anatomical targets. Both liver and hepatic stimulation experiments were performed with portions of the stimulation ultrasound beam extending slightly beyond the target organ (approximately, 2 mm beyond the organ for splenic stimulation and <0.5 mm for hepatic stimulation). Application of new ultrasound transducer technology may be utilized in future experiments to increase the level of stimulus precision^48–50^. Still, the data herein represents the first report in which a targeted ultrasound tool enabled stimulation of multiple points within a single organ (i.e., stimulation of the porta hepatis region versus right or left lobe, Fig. 6), and this initial experiment has already yielded an observation of differential effects based on stimulus location. The data herein also represents the first time that a neuromodulation technique has been used to separately attenuate LPS-induced cytokine versus hyperglycemic effects, showing specificity beyond that available with cervical VNS (Fig. 5). These initial results suggest that further investment and research into the use of ultrasound for peripheral neuromodulation is justified and will require increased collaboration between ultrasound device engineers and neuroscientists.

The results herein also add data to the continued debate around the exact mechanism of ultrasound in neuromodulation experiments^23–30,51^. Supplementary Table 1 provides additional detail comparing the ultrasound parameters used herein to standard clinical measures (MI (mechanical index) and TI (thermal index)) used to assess the potential for heating (TI) or cavitation-based (MI) bioeffects of ultrasound^52^. The ultrasound parameters utilized in the above experiment remain below those expected to heat tissue to temperatures currently known to activate heat-sensitive ion channels^16,53^ or produce cavitation, in agreement with previous reports that have suggested a direct mechanical activation of brain or neural tissue (or surrounding/supporting cell and tissue structures)^29^. The reserpine/catecholamine depletion experiments shown herein also suggest a nerve-mediated mechanism of ultrasound neuromodulation in peripheral organs; however, the results shown above do not specifically identify the cellular or molecular components associated with transduction of the ultrasound energy. Further studies of ultrasound stimulation in in vitro models of organs or organoids (under direct microscopic observation) will be necessary to elucidate the specific molecular transduction pathway of the ultrasound stimulus.

Future translation to the clinic will also come with challenges. As shown in this manuscript, ultrasound parameters shown to provide neuromodulatory effects fall within the range of those currently utilized in the clinic (Supplementary Table 1). However, human organs are larger than the pre-clinical organ targets studied herein, and similar clinical “dosing” studies (as shown herein in pre-clinical model data above, and Supplementary Figures 8–10) will be needed to determine the effect of precision ultrasound stimulation in human anatomy. In addition, there will exist heterogeneity in human patient populations, including body mass index, bone and skeleton size and shape, and nerve/organ location. The tools utilized to test ultrasound-based neuromodulation in the clinic will require the sophistication to adjust stimulation targeting and parameters, such that each patient will receive a known and controlled ultrasound “dose”. Our team is currently designing and building the tools required to achieve precision neuromodulation in both additional pre-clinical models of disease (including models of sepsis, obesity, and diabetes) and within clinical environments. These on-going studies will further test for broad application of non-invasive U/S stimulation to a variety of anatomical and organ targets and determine if these tools translate to the application in a wide range of health disorders.

## Methods

### Focused ultrasound (FUS) probe set-up and characterization

A block diagram of the focused ultrasound (FUS) system is shown in Supplementary Figure 1. The system consists of a 1.1 MHz, High Intensity Focused Ultrasound (HIFU) transducer (Sonic Concepts H106), a matching network (Sonic Concepts), an RF power amplifier (ENI 350L) and a function generator (Agilent 33120A). The 70-mm-diameter HIFU transducer has a spherical face with a 65-mm radius of curvature. It has a 20-mm-diameter hole in the center into which an imaging transducer can be inserted. The transducer depth of focus is 65 mm. The numerically simulated pressure profile has a full width at half amplitude of 1.8 mm laterally and 12 mm in the depth direction. The HIFU transducer is acoustically coupled to the animal through a 6-cm-tall plastic cone filled with degassed water.

The function generator produces a pulsed sinusoidal waveform, shown schematically in Supplementary Figure 2. This pulsed sinusoidal waveform is amplified by the RF power amplifier and sent to the impedance-matching network connected to the HIFU transducer. For most of the animal experiments, the pulse center frequency was 1.1 MHz, the pulse repetition period was 0.5 ms (corresponding to a pulse repetition frequency of 2000 Hz); the pulse amplitude and pulse length varied. Supplementary Table 1 lists the combinations of pulse amplitude and length that were used in the experiments; the third column lists the peak ultrasound pressure at the focus derived from the pulse voltage amplitude.

The voltage-to-pressure calibration of the HIFU transducer was performed in degassed water using a needle hydrophone (ONDA HNA-0400). The HIFU transducer was driven by a 100-cycle sinusoidal voltage waveform. To locate the position of peak pressure, the hydrophone was scanned in a neighborhood of the nominal transducer focus point in 0.1 mm steps in the lateral plane and in 0.2 steps in the depth direction. Supplementary Figure 3 shows a scan through a plane at the depth of focus. For driving voltages below 60 V, the nonlinearity of water was small, i.e., the maximum negative pressure and the maximum positive pressure were nearly equal, and the pressure varied linearly with driving voltage.

### Ultrasound targeting for organ-specific neuromodulation

A Vivid E9 ultrasound system (GE Healthcare) or an 11L probe (GE Healthcare) were used for the ultrasound scan before neuromodulation started. Supplementary Figure 4 shows the images of the rat spleen (Supplementary Figure 4A) and rat liver (Supplementary Figure 4B). The HIFU transducer was positioned on the target area based on this initial image. Another ultrasound scan was also performed using a smaller imaging probe (3S, GE Healthcare), which was placed in the opening of the HIFU transducer (Supplementary Figure 4C). The imaging beam of the 3S probe was aligned with the U/S beam. Therefore, one could confirm that the U/S beam was targeted at the region of interest using an image of the targeted organ (visualized on the Vivid E9). After the organ of interest was identified, the transducer position was marked on the animal’s skin and the HIFU transducer was positioned on the marked area, ultrasound stand-offs were utilized to adjust depth for the appropriate organ target.

### Animal models and ultrasound stimulation protocol

Adult male Sprague–Dawley rats 8–12 weeks old (250–300 g; Charles River Laboratories) were housed at 25 °C on a 12-h light/dark cycle and acclimatized for 1 week, with handling, before experiments were conducted to minimize potential confounding measures due to stress response. Water and regular rodent chow were available ad libitum. Experiments were performed under protocols approved by the Institutional Animal Care and Use Committee of GE Global Research.

Endotoxin (lipopolysaccharide (LPS) from *Escherichia coli*, 0111: B4; Sigma–Aldrich) was used to produce a significant state of inflammation and metabolic dysfunction (e.g., hyperglycemia and insulin resistance) in naive adult Sprague–Dawley rats. LPS was administered to animals (10 mg/kg), which corresponds to an approximate LD75 dose, via intraperitoneal (IP) injection causing significant elevation in concentrations of TNF, circulating glucose, and insulin; these concentrations peak in 4 h but remain elevated as compared to control for up to 8 h post injection. After LPS administration, spleen, liver, hypothalamic, hippocampal, and blood samples were harvested at 60 min for most studies; at 1, 2, and 3 h for kinetic studies (Fig. 2e) and at 0.5, 1, 2, 4, 8, 24, and 48 h for duration studies (Fig. 2g, h). Spleen and liver samples were prepared as described below. Samples were analyzed by enzyme-linked immunosorbent assay (ELISA) for changes in cytokine (Bio-Plex Pro; Bio-Rad), tumor necrosis factor alpha (TNF) (Lifespan) and acetylcholine (Lifespan) concentration as described below. Catecholamine concentrations were assessed using high performance liquid chromatography (HPLC) detection and ELISA (Rocky Mountain Diagnostic) analysis as described below.

The link between LPS and insulin resistance is well detailed in the literature with hyperglycemia and hyperinsulinemia frequently observed during bacterial infection as an indicator of poor clinical outcome in patients. Thus, to follow the effects of LPS and US treatment on blood glucose and insulin levels, blood samples were obtained from the tail vein at 0, 15, 30, and 60 min after LPS injection. Blood glucose concentrations were measured by a OneTouch Elite glucometer (LifeScan; Johnson & Johnson). Insulin concentrations in plasma, obtained from blood, were determined using an ELISA kit (Crystal Chem). Signal transduction changes were measured by assessment of key biomarkers including: p38, p7056k, Akt, GSK3B, c-Src, NF-κβ, SOCS3, IRS-1, NPY, and POMC in liver, muscle, cardiac, and hypothalamic tissue samples.

### Mice

All mice experiments were performed under protocols approved by the Institutional Animal Care and Use Committee of the Feinstein Institute for Medical Research, Northwell Health System. Animals were housed at 25 °C on a 12-h light/dark cycle, and acclimatized for at least 1 week before conducting experiments. Water and regular rodent chow were available ad libitum. Wild type C57black/6 mice, nude (nu/nu) mice, α7 nicotinic receptor knock out mice (B6.129S7-Chrna7tm1Bay, number 003232), ChAT-floxed (B6.129-Chattm1Jrs/J), and mice expressing Cre recombinase under the control of the endogenous CD4 promoter (CD4-Cre), 8–12 weeks old (20–25 g) were purchased from The Jackson Laboratory (Bar Harbor, ME, USA). ChAT-floxed and CD4-Cre mice were crossed to generate mice genetically devoid of ChAT in the CD4+ population.

### Catecholamine depletion

Reserpine (Sigma–Aldrich) dissolved in glacial acetic acid (Sigma–Aldrich) was diluted with sterile saline to a final glacial acetic acid concentration of 0.5%. Mice received intraperitoneal administration of reserpine (10 mg/kg) reserpine 24 h before the beginning of experiments.

### Ultrasound stimulation protocol

The protocol used for ultrasound neuromodulation was as follows. Animals were anesthetized with 2–4% isoflurane. The animal was laid on a water circulating warming pad to prevent hyperthermia during the procedure. The region above the designated point for U/S stimulus (nerve of interest) was shaved with a disposable razor and animal clippers prior to stimulation. A Vivid E9 Diagnostic imaging ultrasound system was used to identify the region of interest as follows. Liver: the porta hepatis as indicated by Doppler identification of the hepatic portal vein. Spleen: visual identification of the spleen by diagnostic ultrasound. Location of stimuli was maintained along the splenic axis as identified. The area was marked with a permanent marker for later identification. Ultrasound stimulation was applied using a research FUS system. The U/S probe was placed at the designated area of interest identified by the diagnostic ultrasound probe (Supplementary Figures 1 and 4). An U/S stimulus was then applied with total duration of a single stimulus not surpassing a single 1 min pulse. At no point was the energy allowed to reach levels associated with thermal damage and ablation/cavitation (35 W/cm^2^ for ablation/cavitation). LPS (10 mg/kg) was then injected IP (for acute/kinetic studies). Alternatively, for duration of effect, LPS was not injected here and was instead injected at a later designated time point. Second 1-min US stimuli may then be applied.

The animal was then allowed to incubate under anesthesia, due to the concentration of LPS being equivalent to an LD75 dose, for acute (1-h) and kinetic (varying up to a maximum of 3 h post LPS) studies. After incubation, the animal was euthanized and tissue, blood samples are collected as described below. For duration of effect studies, LPS was not injected at the time of U/S stimulus but rather at a designated delay after the U/S stimuli have been applied (e.g., 0.5, 1, 2, 4, 8, 24, or 48 h). After the delay, the animal was placed into an anesthetic holding chamber and monitored until euthanasia and tissue/fluid collection.

### Tissue harvesting and sample preparation

An incision was made starting at the base of the peritoneal cavity extending up and through to the pleural cavity. Organs (including spleen and liver) were rapidly removed and homogenized in a solution of phosphate-buffered saline (PBS), containing phosphatase (0.2-mM phenylmethylsulfonyl fluoride, 5-µg/mL aprotinin, 1-mM benzamidine, 1-mM sodium orthovanadate, and 2-µM cantharidin) and protease (1-µL to 20 mg of tissue as per Roche Diagnostics) inhibitors. A targeted final concentration of 0.2-g tissue per mL PBS solution was applied in all samples. Blood samples were stored with the anti-coagulant disodium (ethylenedinitrilo)tetraacetic acid (EDTA) to prevent coagulation of samples. Samples were then stored at −80 °C until analysis.

### ELISA analyses

A detailed protocol for ELISA Assays was provided by the respective supplier of the kits: https://www.lsbio.com/elisakits/manualpdf/ls-f24977.pdf, Acetylcholine: http://www.abcam.com/ps/products/65/ab65345/documents/ab65345%20Choline%20Acetylcholine%20Assay%20Kit%20protocol%20v11%20(website).pdf, PI3K Activation Profile (Akt/GSK/p70S6K): https://www.thermofisher.com/order/catalog/product/85-86048-11, SRC: https://www.lsbio.com/elisakits/manualpdf/ls-f11230.pdf, P38 (MAPK): https://www.thermofisher.com/order/catalog/product/85-86022-11.

### HPLC analyses

Serum samples were injected directly into the machine with no pre-treatment. Tissue homogenates were initially homogenized with 0.1-M perchloric acid and centrifuged for 15 min, after which the supernatant was separated, and the sample injected into the HPLC. Catecholamines norepinephrine and epinephrine were analyzed by HPLC with inline ultraviolet detector. The test column used in this analysis was a Supelco Discovery C18 (15-cm × 4.6-mm inside diameter, 5-µm particle size). A biphasic mobile phase comprised of [A] acetonitrile: [B] 50 = mM KH_2_PO_4_, set to pH 3 (with phosphoric acid). The solution was then buffered with 100-mg/L EDTA and 200-mg/L 1-octane-sulfonic acid. Final concentration of mobile phase mixture was set to 5:95, A:B. A flow rate of 1 mL/min was used to improve overall peak resolution while the column was held to a consistent 20 °C to minimize pressure compaction of the column resulting from the viscosity of the utilized mobile phase. The UV detector was maintained at a 254-nm wavelength, which is known to capture the absorption for catecholamines including norepinephrine, epinephrine, and dopamine.

### Immunohistochemistry and histology protocols

Tissue extraction and paraffin block conversion performed as follows. Put tissue (rat brain) into fixative immediately and fix ~24 h in 10% formalin at 4 °C. Process tissue with the following protocol (with vacuum and pressure during each incubation): 70% ethanol, 37 °C, 40 min, 80% ethanol, 37 °C, 40 min, 95% ethanol, 37 °C, 40 min, 95% ethanol, 37 °C, 40 min, 100% ethanol, 37 C, 40 min, 100% ethanol, 37 °C, 40 min, xylene, 37 °C, 40 min, xylene, 37 °C, 40 min, paraffin, 65 °C, 40 min, paraffin, 65 °C, 40 min, paraffin, 65 °C, 40 min. Sample is then left in this paraffin until ready for embedding (not to exceed ~12–18 h).

Embed into Paraffin block for sectioning, allow block to cool/harden before sectioning. Section 5-µm thick, float on 50 °C water bath for collection. Use positively charged slides and try to position the tissue in the same orientation for every slide. Air dry slides. Overnight at room temperature seems to be the best for drying but the slides can be placed on a 40 °C slide warmer to speed up the drying process, but do not leave slides more than an hour on the warmer. Store slides at 4 °C.

Formalin-fixed paraffin-embedded (FFPE) tissue samples (rat brains) were baked at 65 °C for 1 h. Slides were deparaffinized with xylene, rehydrated by decreasing ethanol concentration washes, and then processed for antigen retrieval. A two-step antigen retrieval method was developed specifically for multiplexing with FFPE tissues, which allowed for the use of antibodies with different antigen retrieval conditions to be used together on the same samples. Samples were then incubated in PBS with 0.3% Triton X-100 for 10 min at ambient temperature before blocking against nonspecific binding with 10% (wt/vol) donkey serum and 3% (wt/vol) bovine serum albumin (BSA) in 1× PBS for 45 min at room temperature. Primary antibody c-Fos (Santa Cruz-SC52; sc-166940) was diluted to optimized concentration (5 μg/mL) and applied for 1 h at room temperature in PBS/3% (vol/vol) BSA. Samples were then washed sequentially in PBS, PBS-TritonX-100, and then PBS again for 10 min, each with agitation. In the case of secondary antibody detection, samples were incubated with primary antibody species-specific secondary Donkey IgG conjugated to either Cy3 or Cy5. Slides were then washed as above and stained in DAPI (10 μg/mL) for 5 min, rinsed again in PBS, and then mounted with antifade media for image acquisition. Whole-tissue images were acquired on fluorescence microscope (Olympus IX81) at  ×10 magnification. Autofluorescence, which is typical of FFPE tissues, needs to be properly characterized and separated from target fluorophore signals. We used autofluorescence removal processes, wherein an image of the unstained sample is acquired in addition to the stained image. The unstained and stained images are normalized with respect to their exposure times and the dark pixel value (pixel intensity value at zero exposure time). Each normalized autofluorescence image is then subtracted from the corresponding normalized stained image. We ensured that the same region in the stimulated and control samples were imaged.

### Histological assessment of stimulated tissue

Spleen from stimulated rats and control rats were processed into paraffin blocks as described above. Paraffin-embedded sections were cleared and stained for H&E following standard protocol reported in the literature and scanned on a bright field scanner (Olympus). H&E images were qualitatively assessed for morphological difference and no significant difference was noticed between stimulated and control samples (Supplementary Figure 7).

### Alternate pulse repetition periods and pulse amplitude

Data for blood glucose and/or splenic NE, ACH, and TNF concentrations in ultrasound-stimulated animals (with (Supplementary Figures 8–10) or without (Supplementary Figure 7) LPS injection) using alternative ultrasound-stimulation parameters were compared to those presented in the main text. We evaluated the effect of ultrasound neuromodulation in naive/non-LPS treated animals (Supplementary Figure 7) and animals treated with alternative pulse repetition periods and pulse lengths (summary of results in Supplementary Figures 8–10).

### Electrode-based vagal nerve stimulation (VNS) protocol

Male Sprague–Dawley rats were anesthetized with 2–4% isoflurane. A single incision was made along the neck exposing the cervical portion of the trapezius, sternocleidomastoid and masseter muscles for blunt dissection exposing the left cervical vagus nerve. The microelectrode was placed along the main trunk of the exposed cervical vagus nerve. Electrical stimulation using three settings (5 V, 30 Hz, 2 ms; 5 V, 5 Hz, 2 ms; and 1 V, 5 Hz, 2 ms) was generated using a BIOPAC MP150 module under the control of the AcqKnowledge software (Biopac Systems). Rats underwent 3 min of VNS before and after IP injection of 10-mg/kg LPS. Following injection of 10 mg/kg LPS, a saline-soaked pad was used to hydrate the area and the cervical region was sutured closed to maintain integrity of the physiologic site. Rats were euthanized 60 min after LPS injection as described above, and spleen and blood samples were obtained for TNF determination as described above. In rats subjected to sham surgery, the vagus nerve was exposed, but not touched or manipulated.

### Heart rate monitoring and analysis

Heart rate (during either ultrasound or electrode stimulation experiments) was monitored using a commercial infrared oximeter and physiological monitoring system (Starr Lifesciences) using the manufacturer’s instructions. During the stimulation protocols, the foot clip sensor (provided by the manufacturer) was placed on the footpad of the animal. The animal was allowed to acclimate for at least 5 min prior to measurement, a time point found sufficient for animals to recover to normal heart rate activities and physiological reading in controls. Measurement was recorded before (2-min recording periods), during, and after (2-min recording periods) the stimulation with either the electrical microelectrode or ultrasound probe.

### cFos analysis and other measures of US-induced activation

LPS-U/S stimulated and sham animals were rapidly euthanized, and brains removed and transferred to 10% paraformaldehyde for 24 h, after which they were transferred to a 30% sucrose solution and stored for 4 °C prior to paraffin embedding (detailed in the IHC section above). Coronal section (5–10 µm) were cut by cryostat. Structures were anatomically defined according to an anatomical atlas. Quantification of c-Fos positive cells was counted with a fixed sample window across at least four sections by an experimenter blinded to the treatment conditions associated with each distinct coronal section. Regions of interest were as follows: paraventricular hypothalamic nucleus, ARC, VMN, DMN, LH, and mammillothalamic tract (all structures visible in coronal slices taken between Bregma −2.56 to −3.60 mm). The number of c-Fos positive cells in each group were expressed as a % of cFos+ cells as compared to Sham-stimulated control littermates.

In addition to the hypothalamic specific cFos-staining analysis performed in the main Bregma −11.3 to −14.08 mm, text/figures, the team sectioned and analyzed ultrasound induced activation within the NTS. The NTS is a brainstem nucleus known to harbor purely sensory nuclei (including fibers from the vagus nerve), and project to areas of the brain involved with autonomic regulation (including the hypothalamus; see Supplementary Figure 11 for a description of these neural pathways). The increased staining of the activation specific marker in the ultrasound stimulated animals (Fig. 7) agrees with the other findings (i.e., hypothalamic neurotransmitter and protein data, and MRI data) and suggests ultrasound induction of a nerve-mediated modulation of hypothalamic metabolic control centers. This additional supplemental data (Supplementary Figure 12) suggests that this ultrasound effect is at least in part mediated through direct sensory pathways.

### Diffusion functional MRI

Neuronal activation is typically detected using blood-oxygenation-level-dependent (BOLD) fMRI^54^; brain regions with increased metabolic demand lead to higher cerebral blood flow, an increased supply of oxygenated blood, and decreased gradient echo signal. Sensitivity to the BOLD effect requires the use of fast gradient echo acquisitions; this causes undesired signal loss in brain areas next to air pockets, such as sinuses and ear canals, and hinders detection of neuronal activation near those specific brain areas. Alternatively, to minimize signal loss in areas characterized by large field inhomogeneities, spin echo (or double spin echo) diffusion-weighted imaging (DWI) can be used for detecting neuronal activation^44–46^ or nerve activation driven by external neuromodulators^47^. In DWI-fMRI, a volume increase in the slow-diffusing, presumably intracellular, water pool or an increase in water diffusion (or ADC) are assigned physical changes caused by neuronal activation.

Ten Sprague–Dawley rats were anesthetized using 3% Isoflurane and placed supine, with their heads inserted in a birdcage coil. The abdomen region was coupled through a gel/water filled cone to an MR-compatible U/S probe (*f* = 1.47 MHz), focusing on the porta hepatis, a liver region known to contain glucose sensitive neurons. Supplementary Figure 13a depicts a schematic of the experimental setup, including the US probe connected to RF amplifier/signal generator.

Data were acquired on a 3T scanner (MR750, GE Healthcare). An SPGR T1 acquisition was followed by six blocks of DWI images, with a TE/TR of 82/3400 ms, using 3/4 averages for the *b* = 0/*b* = 1000 s/mm^2^ and 0.6/1-mm in-plane/out-of-plane spatial resolution. An additional reverse polarity DWI acquisition was acquired for distortion correction purposes^55^. Following the LPS injection, the first US treatment, a wait time and the second US treatment, another 6 blocks of DWI images were acquired. Each ultrasound treatment lasted 60 s, during which square wave pulses were applied at 150/350-μs on/off periods. The sound pressure at the focal point was approximately 3.2 MPa. Supplementary Figure 13b depicts a summary of the experimental timing. This protocol was applied to 6 rats; for the remaining 4, the last DWI blocks immediately followed the LPS injection, with no US treatment.

A cross-correlation coefficient (*ccc*) between the T1 images and the (distortion-corrected) *b* = 0 DWI images of at least 0.5 was used to identify slices to be used for further analysis. ADCs were calculated for the pre- and post-treatment images; pre- and post-treatment image data were pooled together for statistical analysis. A rigid registration between the T1 images and a rat atlas was used to determine regions in which pixel-by-pixel *t*-tests indicated significant changes. The registration transformation from the T1 and atlas images was applied to the distortion-corrected DWI and ADC images.

Supplementary Figure 14 shows an example of the T1/*b* = 0 DWI acquisition in one rat after distortion correction; only red-highlighted slices met *ccc* > 0.5 and were kept for statistical analysis. To follow the effects of LPS and US treatment on blood glucose level, blood samples were obtained from the tail vein immediately before the first MRI scan and at 30 min after LPS injection; blood glucose concentration was measured by a OneTouch Elite glucometer (LifeScan; Johnson & Johnson).

Figure 7b shows an example overlay between the activation maps/SPGR volume (left) and the atlas/SPGR volume (right). Note the ADC change in both PVNs of the hypothalamus (red arrows, left image), consistent with ultrasound-induced neuromodulation. Figure 7d summarizes the results in a bar graph and Supplementary Table 2 details the explicit results in all 10 animals. Three of six rats showed significant neuromodulation in the PVNs; none of the control animals showed such change in neural activity. Furthermore, the hyperglycemia observed in the non-US-treated animals was not observed in the US-treated animals.

## Supplementary information

Supplementary Information

## Data Availability

Data generated or analyzed during this study were made available within the published article where possible (and its Supplementary Information files); any dataset generated during and/or analyzed during the current study may also be made available from the corresponding author on reasonable request. Microscopy images utilized in the manuscript have been made available at https://datadryad.org. (10.5061/dryad.md888qr).
