# Author Correction: Noninvasive sub-organ ultrasound stimulation for targeted neuromodulation

**DOI:** 10.1038/s41467-020-15011-7

**Published:** 2020-03-09

**Authors:** Victoria Cotero, Ying Fan, Tea Tsaava, Adam M. Kressel, Ileana Hancu, Paul Fitzgerald, Kirk Wallace, Sireesha Kaanumalle, John Graf, Wayne Rigby, Tzu-Jen Kao, Jeanette Roberts, Chitresh Bhushan, Suresh Joel, Thomas R. Coleman, Stavros Zanos, Kevin J. Tracey, Jeffrey Ashe, Sangeeta S. Chavan, Christopher Puleo

**Affiliations:** 1 GE Global Research Center, 1 Research Circle, Niskayuna, NY 12309 USA; 2 Feinstein Institute for Medical Research, 350 Community Drive, Manhasset, NY 11020 USA

**Keywords:** Ultrasound, Peripheral nervous system, Inflammation

Correction to: *Nature Communications* 10.1038/s41467-019-08750-9, published online 12 March 2019.

This article contained an error in the ordinate axis in Fig. 2d, where the units were reported as nmol/L. The unit should have been reported as pg/ml. This has now been corrected in both the PDF and HTML versions of the Article.

