# Supplementary material for: Noninvasive sub-organ ultrasound stimulation for targeted neuromodulation
Source: Nat Commun. 2019 Mar 12;10:952. doi: 10.1038/s41467-019-08750-9 (PMC6414607; doi:10.1038/s41467-019-08750-9)
Supplement: Supplementary file 1 — Supplementary Information [file 41467_2019_8750_MOESM1_ESM.pdf]

Supplemental Figures for:

Noninvasive Sub-Organ Ultrasound Stimulation for Targeted Neuromodulation

Victoria Cotero et. al.

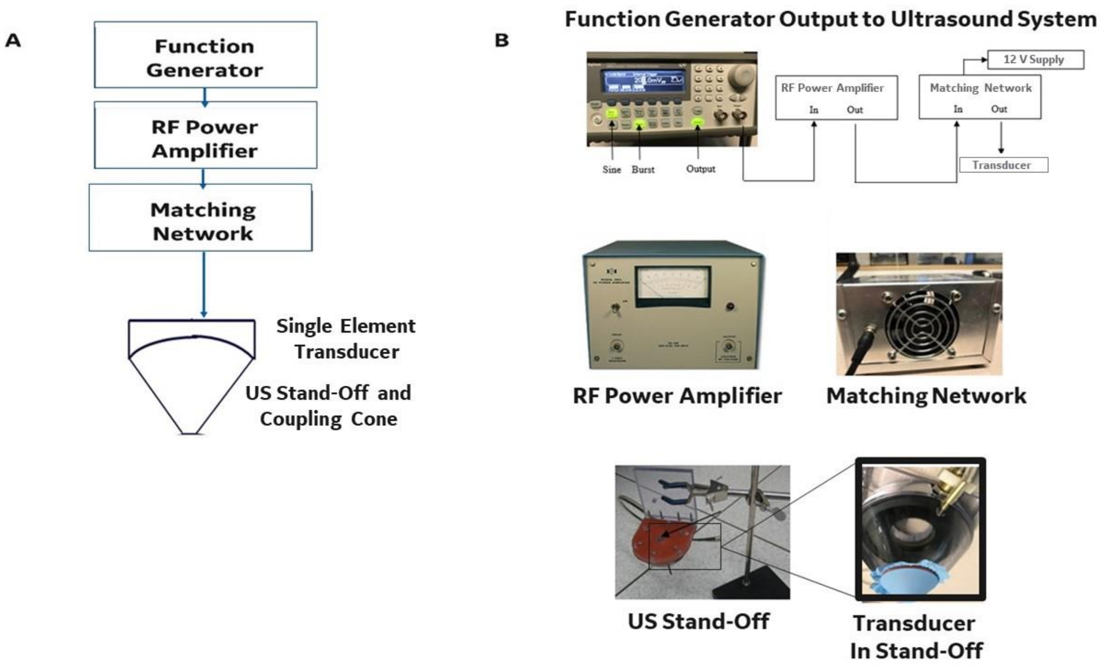

Supplementary Figure 1. Schematic and Images of the Experimental Ultrasound (US) Neuromodulation Setup. A. The system consists of a 1.1 MHz Single Element Focused Ultrasound transducer (Sonic Concepts H106), a matching network (Sonic Concepts), an RF power amplifier (ENI 350L) and a function generator (Agilent 33120A). The 70-mm-diameter transducer has a spherical face with a 65-mm radius of curvature. It has a 20-mm-diameter hole in the center into which an imaging transducer can be inserted. The transducer depth of focus is 65 mm. The numerically simulated pressure profile has a full width at half maximum amplitude (FWHM) of 1.8 mm laterally and 12 mm in the depth direction. The transducer is acoustically coupled to the animal through a 6-cm-tall plastic cone filled with degassed water (US stand-off and coupling cone). **B.** Images of the components described above show the function generator, which produces a pulsed sinusoidal waveform, shown schematically in fig. S2. This pulsed sinusoidal waveform is amplified by the RF power amplifier and sent to the impedance-matching network connected to the transducer. For most of the animal experiments, the pulse center frequency was 1.1 MHz, the pulse repetition period was 0.5 ms (corresponding to a pulse repetition frequency of 2000 Hz); the pulse amplitude and pulse length varied. Table S1 (below) lists the combinations of pulse amplitude and length that were used in the splenic stimulation experiments.

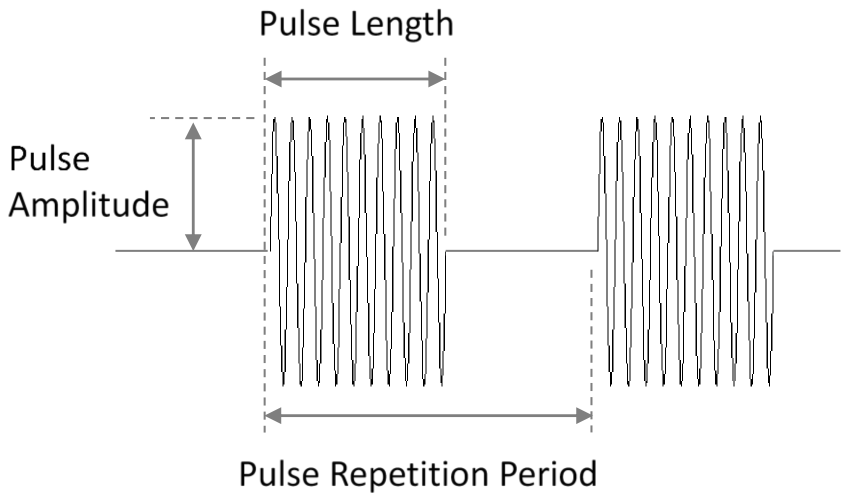

Supplementary Figure 2. Schematic of the Pulsed Sinusoid Waveform Used to Drive US Neuromodulation. The function generator (described in Supplementary Figure 1) produces a pulsed sinusoidal waveform, as shown in the above schematic. This pulsed sinusoidal waveform is amplified by the RF power amplifier and sent to the impedance-matching network connected to the transducer to drive transducer output.

AF (MHz)	PRP (mS)	Duty Cycle	Pulse Length (uS)	Stim Time	Input Volts (Vpeak)	Peak Pos. Pressure (Mpa)	Peak Neg. Pressure (Mpa)	Pulse Average Intensity (W/cm2)	Temporal Average Intensity (mW/cm	MI	TI
1.10	0.50	0.27	136.36	1 min	62.00	1.72	1.52	98.61	26895	1.13	1.91
1.10	0.50	0.27	136.36	1 min	46.50	1.27	1.15	53.76	14663	0.86	1.04
1.10	0.50	0.27	136.36	1 min	31.00	0.83	0.78	22.96	6263	0.58	0.44
1.10	0.50	0.27	136.36	1 min	15.50	0.41	0.39	5.60	1528	0.29	0.11
1.10	0.50	0.27	136.36	1 min	9.60	0.25	0.25	2.08	568	0.18	0.04
1.10	0.50	0.27	136.36	1 min	7.75	0.20	0.20	1.33	364	0.15	0.03
1.10	0.50	0.27	136.36	1 min	5.00	0.13	0.13	0.56	154	0.10	0.01
1.10	0.50	0.27	136.36	1 min	0.50	0.01	0.01	0.01	1.54	0.01	0.00

Supplementary Table 1. List of Ultrasound Neuromodulation Parameters used in the Splenic Study. AF is the acoustic frequency of the transducer output; PRP is the pulse repetition period of ultrasound stimulus (as shown above in Supplementary Figure 2); duty cycle is the percent of time that the ultrasound stimulus is on within each pulse repetition period; pulse length is the total burst duration (as shown above in Supplementary Figure 2), stim time is the amount of time the ultrasound stimulus was applied to the target location for treatment, input volts is the required input voltage to the transducer to achieve the stimulus intensities listed, MI is the calculated Mechanical Index (a measure of the potential for bioeffects due to cavitation; FDA limit for diagnostic imaging is 1.9), TI (thermal index in soft tissue) is the calculated Thermal Index (a measure of the potential for bioeffects due to ultrasound induced temperature increase and an estimate (in degree C) of the temperature increase at the focal spot; diagnostic ultrasound is typically performed at TI less than 2).

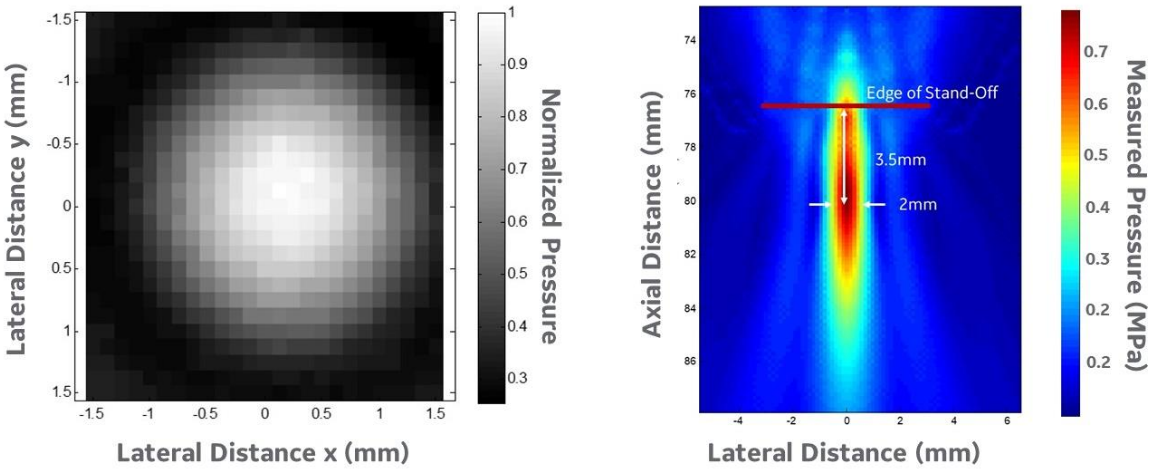

Supplementary Figure 3. Measured Normalized Maximum Pressure as a Function of Lateral Position in Plane at the Depth of Focus. **Left**. The voltage-to-pressure calibration (results shown in Supplementary Table 1) of the single element transducer was performed in degassed water using a needle hydrophone (ONDA HNA-0400). The single element transducer was driven by a 100-cycle sinusoidal voltage waveform. To locate the position of peak pressure, the hydrophone was scanned in a neighborhood of the nominal transducer focus point in 0.1 mm steps in the lateral plane and in 0.2 steps in the depth direction. The figure shows a scan through a plane at the depth of focus. For driving voltages below 60 V, the nonlinearity of water was small, i.e., the maximum negative pressure and the maximum positive pressure were nearly equal, and the pressure varied linearly with driving voltage. **Right.** The same procedure describe above was performed in the axial direction to provide information on the depth of the focused ultrasound beam. A line is drawn (red) to show the edge of the stand-off/coupling cone; this is the level of contact with the skin.

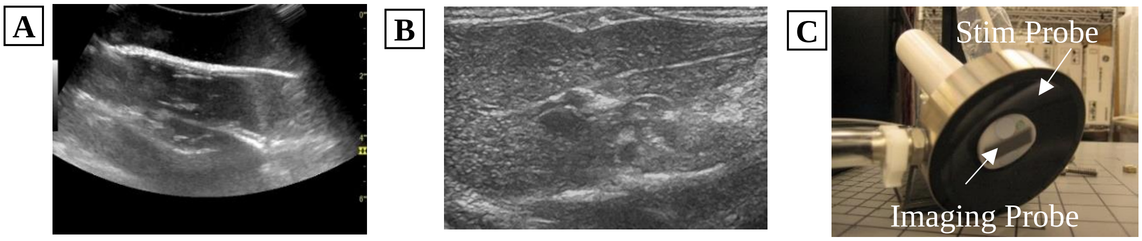

Supplementary Figure 4. Example Images used to Locate Splenic and Hepatic Target Stimulation/Neuromodulation Sites. A Vivid E9 ultrasound system (GE Healthcare) or an 11L probe (GE Healthcare) were used for the ultrasound scan before neuromodulation started. The figure shows example images of rat spleen (A) and rat liver (B) used to locate stimulation targets. A single element transducer (C) was positioned on the target area based on this initial image. In some cases, another ultrasound scan was also performed using a smaller imaging probe (3S, (GE Healthcare), which was placed in the opening of the single element transducer (example shown in C). In this case, the imaging beam was aligned with the single element transducer/beam. Therefore, one could confirm that the single element transducer beam was targeted at the region of interest using an image of the targeted organ. After the organ of interest was identified, the transducer position was also marked on the animal's skin to ensure positioning of the single element transducer on the marked area.

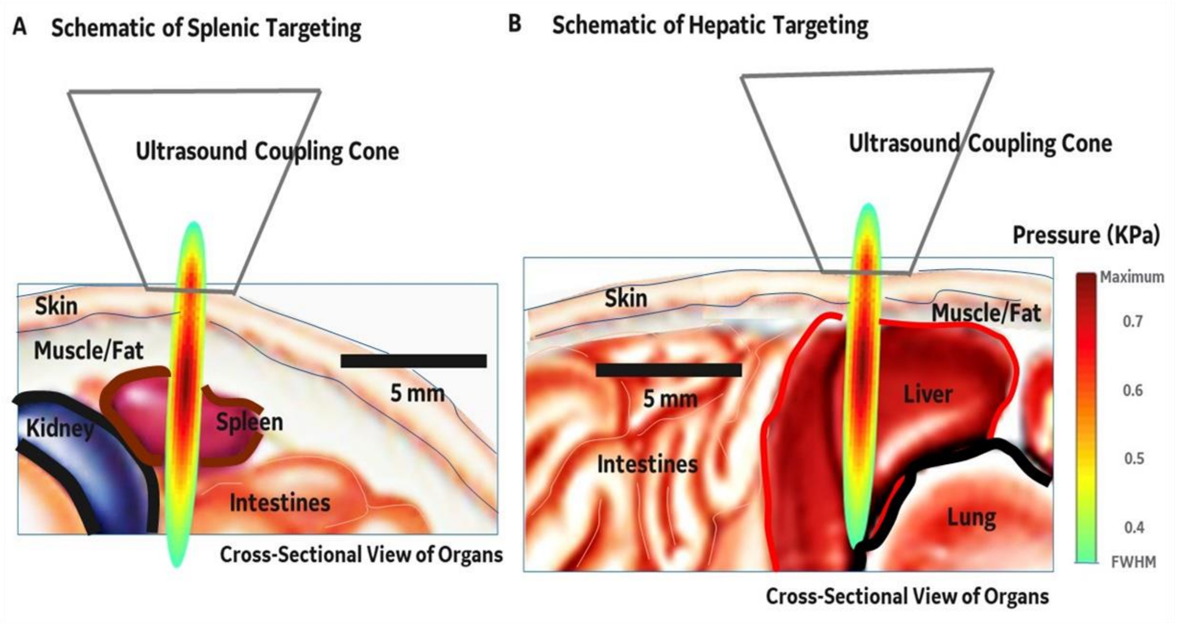

Supplementary Figure 5. Schematic diagram of the level of targeting in the spleen (A) and liver (B) experiments. The schematics were made using data and scale/dimensions from Supplementary Figures 3 and 4. **A.** A schematic of the measured distances to the splenic target is overlaid with the estimated ultrasound pressure within the ultrasound beam (using the 0.83 MPa pressure setting utilized throughout most of the manuscript). Although more targeted than previous reports in the literature, not all ultrasound energy in our experiments is focused within the spleen; however, the experiment was designed to restrict any pressure above the full width half maximum (FWHM) to fall within 2 mm of the splenic target. **B.** A schematic of the measured distances to the liver target is overlaid with the estimated ultrasound pressure within the ultrasound beam (using the 0.83 MPa pressure setting utilized throughout most of the manuscript). As the liver is larger than the spleen, it is expected that (when aligned properly as described above) there is not a significant amount of ultrasound pressure (above FWHM) penetrating beyond the liver. In corroboration, the team showed in manuscript figure 6B that this level of targeting was adequate to demonstrate anatomical specificity of the ultrasound effect (when targeting the porta hepatis versus the surrounding hepatic lobes).

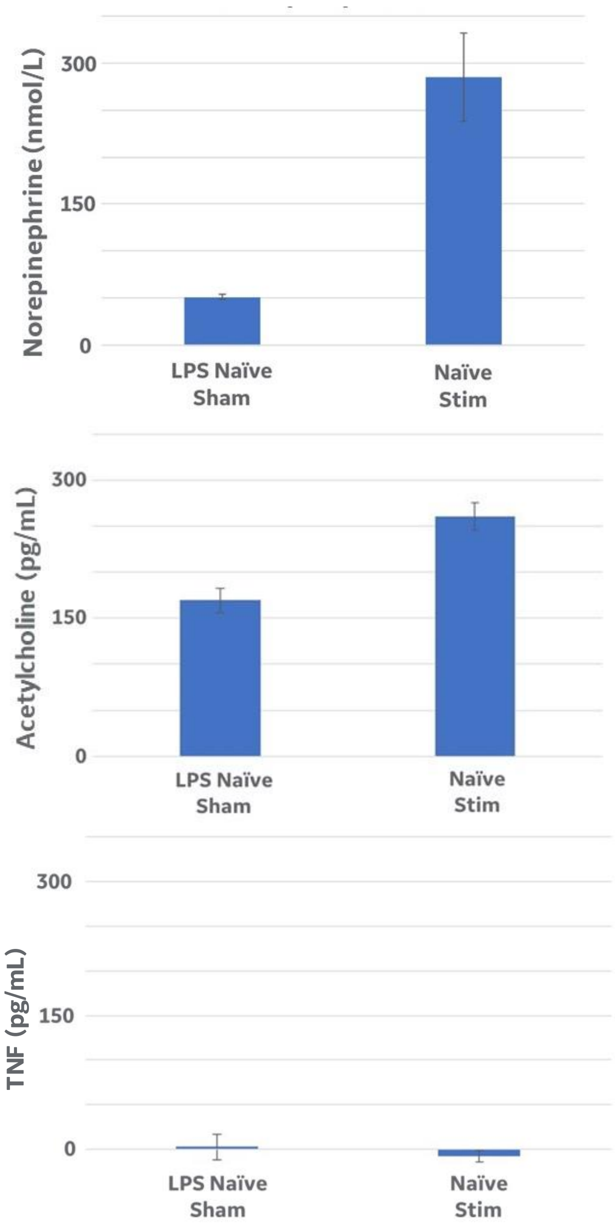

Supplementary Figure 6. Splenic US Neuromodulation of the CAP in LPS-naïve animals. Splenic ultrasound neuromodulation was repeated at the optimal parameters found in figure 2 of the main manuscript (0.83 MPa setting) in LPS-naïve animals. Despite the lack of LPS stimulation, norepinephrine levels (top) were increased upon ultrasound stimulation (suggesting that the ultrasound stimulations effect on nerve activity is not dependent on the LPS stimulus). In agreement with current understanding of the cholinergic anti-inflammatory pathway, the ACH (middle) also increased within the spleen. However, in the naïve animals (in which pre-ultrasound stimulation levels of TNF are already at physiological baseline or at levels below the limit of detection of standard Elisa assays) there is not a measured change in TNF levels (unlike the LPS-stimulated experiments in figure 2 that show a significant CAP induced reduction in TNF when ultrasound is used to modulate CAP signaling in the inflamed state). Error bars on the charts represent standard error across n=5 replicates.

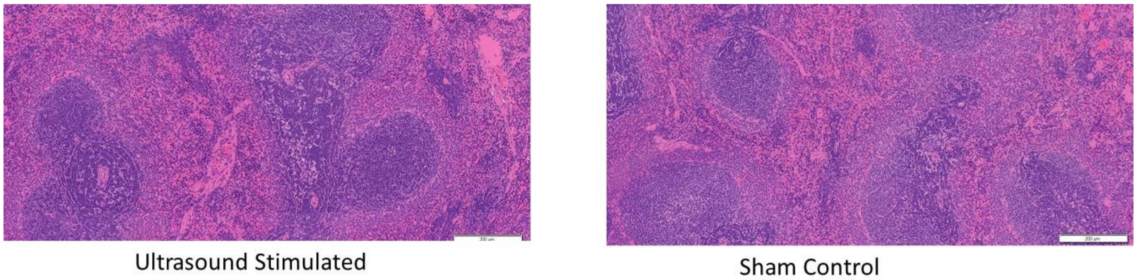

Supplementary Figure 7. Example Histological Sections from Rat Spleens with or without Ultrasound Stimulation. Spleen from stimulated rats (left) and control rats (rat) were processed into paraffin blocks as described in methods. Paraffin-embedded sections were cleared and stained for H&E following standard protocol reported in the literature and scanned on a bright field scanner (Olympus). H&E images were qualitatively assessed for morphological difference and no significant difference was noticed between stimulated and control samples. Scale Bar = 200 microns

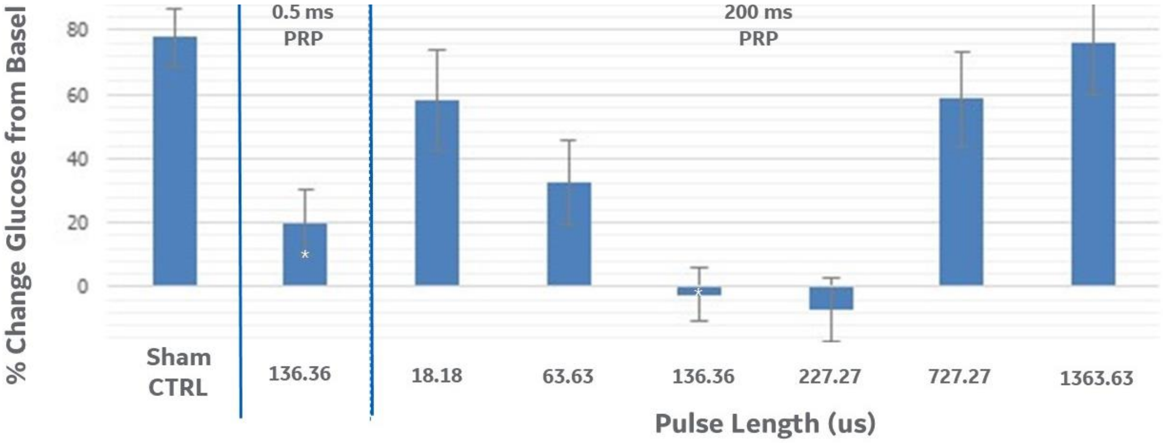

Supplementary Figure 8. Ultrasound PRP and Pulse Length Modification and Effect on Ultrasound Attenuation of Hyperglycemia. The effect of hepatic ultrasound neuromodulation in attenuation of the hyperglycemic effect of LPS was first repeated at the "optimal" splenic stimulation parameters, as found in manuscript figure 2 and used in manuscript figure 6 (i.e. 1.1 MHz, 0.5 ms PRP, 136.36 us pulse length, and 0.83 MPa; data point shown in between vertical bars). PRP was then varied, and results at a more optimal PRP (200 ms) are shown here. Pulse length was varied at this more optimal PRP from 18.18 us – 1336.36 us, while keeping the peak positive pressure constant at 0.83 MPa. Ultrasound stimulus duration was also kept constant (2- minute treatment as shown in the manuscript); thus, this data shows that significantly fewer pulses can be applied to the hepatic stimulus area (i.e. PRP reduction from 0.5 ms to 200 ms) while retaining the effect on blood glucose. However, the required pulse length remains similar to those found in the previous experiments in both the spleen and liver (figure 2 and 6 in the manuscript). Each data bar within this graph represents average values with standard error from n=5 animals.

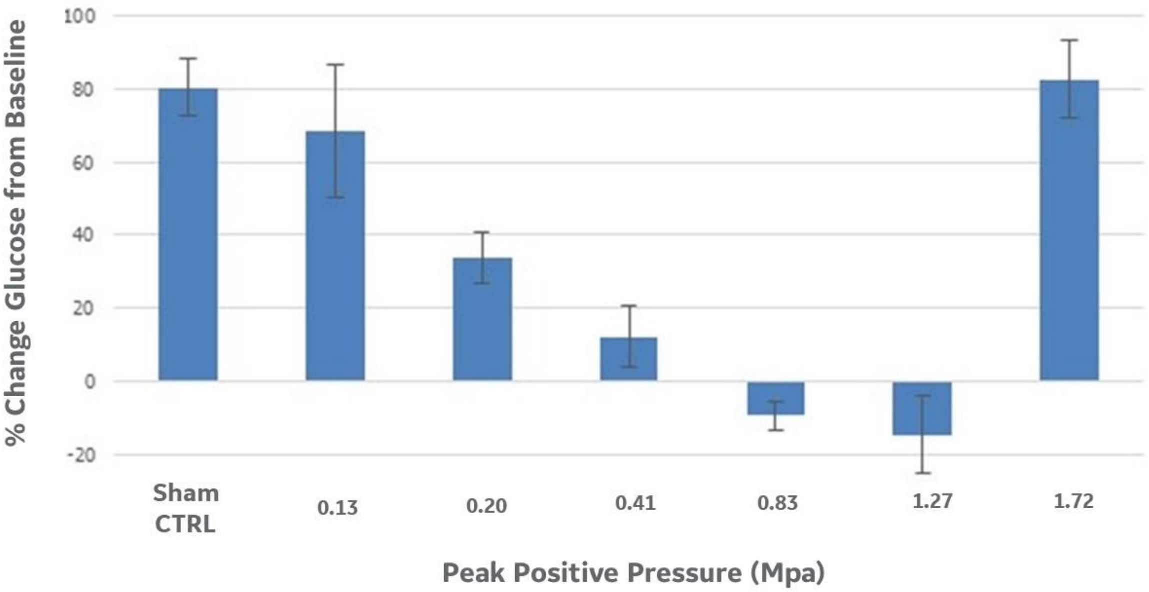

Supplementary Figure 9. Ultrasound Peak Pressure Modification and Effect on Attenuation of Hyperglycemia. Supplemental Figure 9 shows that even though PRP can be reduced (i.e. the total number of pulses provided in a given 2-minute stimulus can be reduced and still retain effectiveness as shown in figure Supplementary Figure 8), the peak pressure within the pulse must be kept similar to those found in the 0.5 ms PRP experiments outlined in the manuscript text (i.e. the pressures within each pulse cannot be reduced without losing effectiveness of the ultrasound neuromodulation). The data shows the level of attenuation of LPS-induced hyperglycemia using hepatic ultrasound stimulation at 1.1 MHz, 200 ms PRP, 136.36 us pulse length, and varying peak positive pressures from 0.13 – 1.72 MPa). Each data bar within this graph represents average values with standard error from n=5 animals.

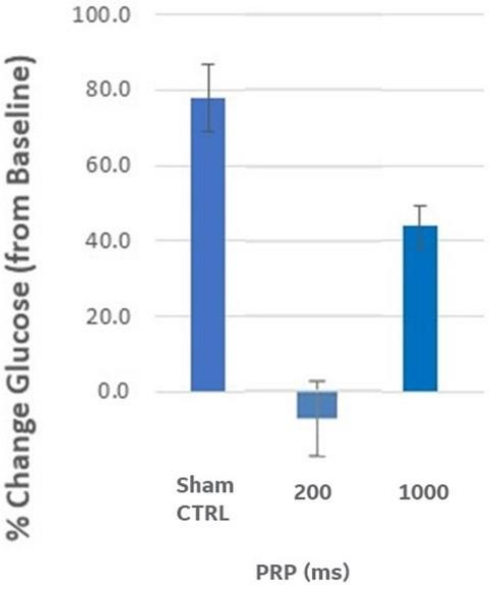

Supplementary Figure 10. Additional ultrasound parameter (PRP) modifications. Figure 10 is a check to see that the 200 ms PRP stimulation parameter (data shown in Supplementary Figure 8) cannot be further reduced. The data shows the effect of hepatic ultrasound neuromodulation on attenuation of LPS-induced hyperglycemia using ultrasound stimulation at 1.1 MHz, 136.36 us pulse length, 0.83 MPa peak positive pressure, and a PRP of either 200 or 1000 ms. Each data bar within this graph represents average values with standard error from n=5 animals.

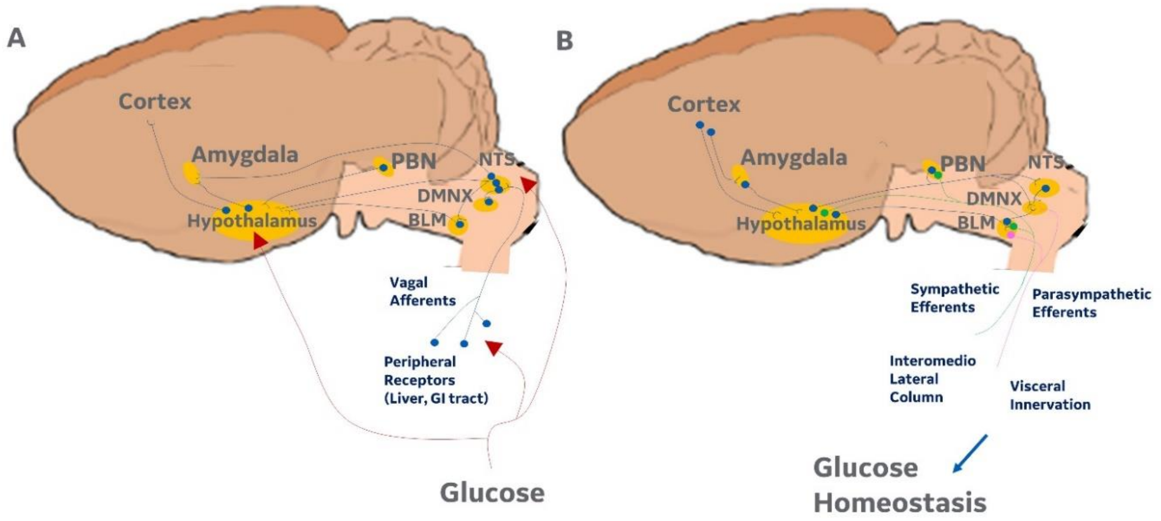

Supplementary Figure 11. Schematic Diagram of Important Afferent (left) versus Efferent (right) Neural Pathways. These are important pathways Involved with Glucose Regulation and Homeostasis (Summarized from information contained in 47,48)**.** (left) Afferent vagal pathways are known to be activated by gut or hepatoportal sensory neurons, and project to the nucleus tractus solitaris (NTS). Glucose sensing neurons are also present in the dorsal vagal complex (consisting of the NTS, the area postrema (AP), and the dorsal motor nucleus of the vagus nerve (DMNX)), and the basal hypothalamus. This enables glucose and nutrient concentration information to be transmitted to the hypothalamus either directly (through local sensory neurons) or indirectly from the peripheral sensors (via the basolateral medulla (BLM) or the parabrachial nucleus (PBN)). At the hypothalamus, all the sensory information in integrated to enable an appropriate response, which requires interaction between the hypothalamus and other cerebral structures (such as the amygdala and cortex). **B.** Efferent pathways activated in response to blood glucose concentration fluctuations project to both sympathetic and parasympathetic neurons. Sympathetic pathways project from the hypothalamus to the intermediolateral cell column, receiving inputs from the PBN and BLM. Parasympathetic pathways project from the BLM, NTS, AP, and DMNX (with modulation via interaction between the hypothalamus and the BLM/DMNX). Current understanding is that sympathetic projections are responsible for stimulating glucagon secretion, inhibiting insulin secretion, activating adrenal secretion of epinephrine, and activation of thermoregulatory brown adipose tissue. Parasympathetic projections are thought to be responsible for insulin secretion and inhibition of hepatic glucose production.

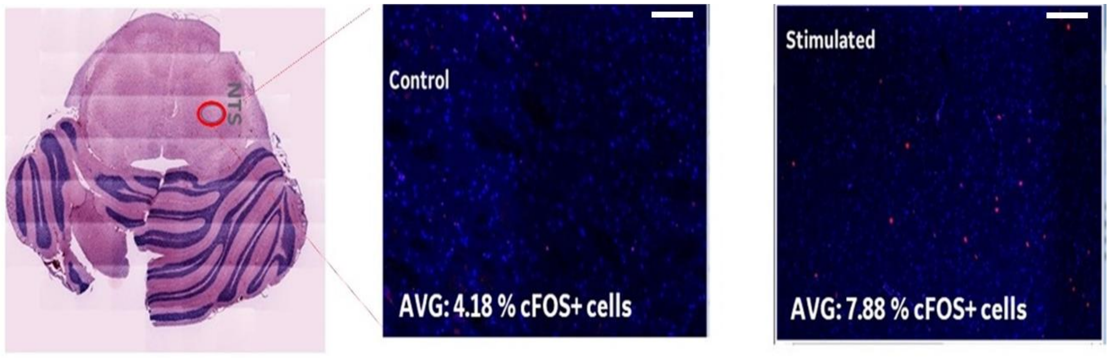

Supplementary Figure 12. Tissue Sections and Immunohistochemistry results from the Nucleus Tractus Solitaris (NTS). (left) An H+E stained section used to identify the location of the NTS (based on anatomical landmarks) in neighboring sections. (right) Immunohistochemistry images showing cFOS expression in the area defined as the NTS in both unstimulated controls (left) and U/S-stimulated animals (right). Average data from three separate control vs. ultrasound stimulated samples are shown in text on each image; the average number of cFOS+ cells increased from 4.18% to 7.88% of the total cells within the NTS in the stimulated samples. Scale Bar = 50 microns

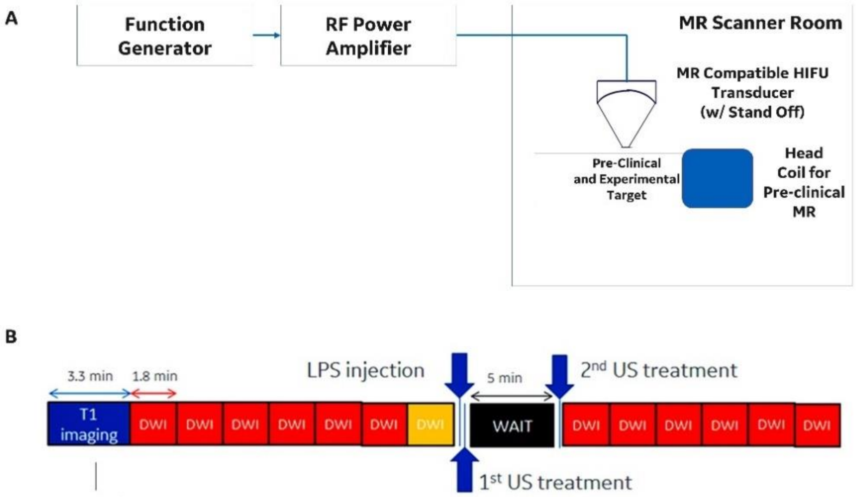

Supplementary Figure 13. Experimental Setup and Protocol for DfMRI based Analysis of Hepatic Ultrasound Neuromodulation. **A.** Ten Sprague-Dawley rats were anesthetized using 3% Isoflurane and placed supine, with their heads inserted in a birdcage coil. The abdomen region was coupled through a gel/water filled cone to an MR-compatible U/S probe (1.47 MHz), focused on the porta hepatis, a liver region previously shown to contain glucose sensitive neurons (as described in the text). The U/S probe was connected to a RF amplifier/signal generator, as described above. The slight modification to the ultrasound parameters (i.e. 1.47 vs. 1.1 MHz in the rest of the experiments within the manuscripts) was necessary, as a separate MR compatible transducer was essential in the DfMRI tests. **B.** Experiments proceeded as described in the text and methods (for U/S neuromodulation of LPS-induced hyperglycemia), with DfMRI procedure performed as follows: Data were acquired on a 3T scanner (MR750, GE Healthcare). An SPGR T1 acquisition was followed by six blocks of DWI images, with a TE/TR of 82/3400 ms, using 3/4 averages for the b=0/b=1000 s/mm^2 and 0.6/1-mm in-plane/out-of-plane spatial resolution. An additional reverse polarity DWI acquisition was acquired for distortion correction purposes (*42*). Following the LPS injection, the first U/S treatment, a wait time and the second U/S treatment, another 6 blocks of DWI images were acquired. Each ultrasound treatment lasted 60 seconds, during which square wave pulses were applied at 150/350-μs on/off periods. The sound pressure at the focal point was approximately 3.2 MPa. This protocol was applied to 6 rats; for the remaining 4, the last DWI blocks immediately followed the LPS injection, with no U/S treatment.

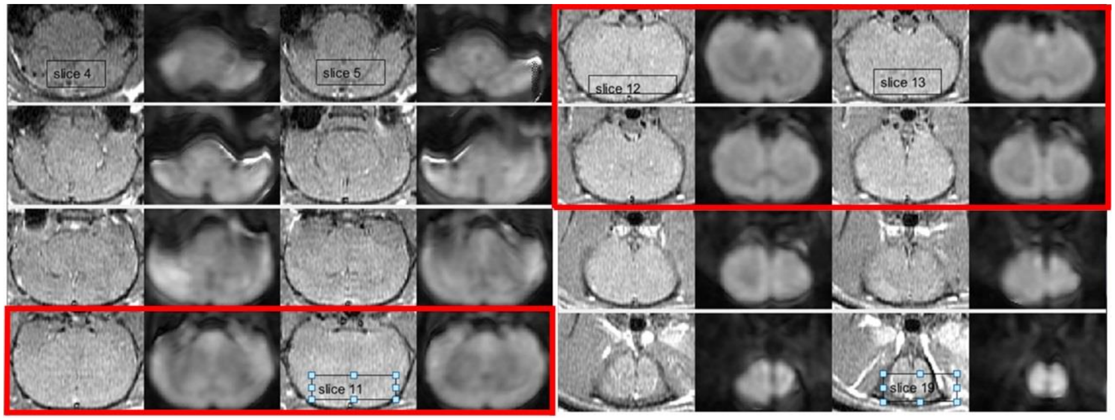

Supplementary Figure 14. Example of T1/b=0 DWI images after distortion correct. After the procedure outlined in Supplementary Figure 13, a cross-correlation coefficient (*ccc*) between T1 images and the (distortion-corrected) b=0 DWI images of at least 0.5 was used to identify slices to be used for further analysis. ADCs were calculated for the pre- and post-treatment images, and then pre- and post-treatment image data were pooled together for statistical analysis. A rigid registration between the T1 images and a rat atlas was used to determine regions in which pixel-by-pixel t-tests indicated significant ADC changes. The registration transformation from the T1 and atlas images was applied to the distortion-corrected DWI and ADC images. This figure shows an example of the T1/b=0 DWI acquisition in one rat after distortion correction (only red-highlighted slices met ccc>0.5 and were kept for statistical analysis).

		Left PVN t-test values*			Right PVN t-test values*			Blood glucose concentration (mg/dL)		
		ROI min.	ROI ave.	ROI std. dev.	ROI min.	ROI ave.	ROI std. dev.	pre-LPS	30-min-post-LPS	change
With U/S stimulation	Rat 1	-29.1	-23.8	6.3	-24.9	-17.9	7.3	N/A	238	N/A
	Rat 2	-3.8	-2.8	1.1	-2.8	-1.6	0.8	N/A	196	N/A
	Rat 3	-7.2	-4.2	2.3	-1.0	0.6	1.4	N/A	204	N/A
	Rat 4	-12.6	-10.6	1.5	-12.1	-10.6	1.2	233	246	13
	Rat 5	-22.0	-19.1	1.9	-22.8	-20.2	2.6	146	171	25
	Rat 6	-3.9	-2.9	0.6	-1.3	-1.3	2.3	124	183	59
	Ave.		-10.6			-8.5		168	206	39
	St. dev.		2.1			2.4		58	30	
Without U/S stimulation	Rat 7	-2.7	-1.3	1.2	-2.1	-0.6	1.2	N/A	440	N/A
	Rat 8	-4.3	-3.6	0.6	-3.8	-2.2	1.3	186	362	176
	Rat 9	-2.6	-1.7	1.7	-0.4	-0.0	0.7	206	263	57
	Rat 10	2.3	2.5	0.6	0.4	2.0	1.0	137	354	217
	Ave.		-1.0			-0.2		176	355	178
	St. dev.		2.6			1.7		36	72	

* t-test values obtained from the comparison of the ADC values within the validated PVN ROIs between the pre- and post-treatment scans.

Supplementary Table 2. T-test Values Obtained through the Comparison of ADC Values within the PVN Regions-of-Interest. Results are shown as comparison between Pre- and Post-Treatment Scans. The table summarizes all results for the DfMRI experiments, where figure 7B in the text/manuscript shows an example overlay between the activation maps/SPGR volume (left) and the atlas/SPGR volume (right; used to located activity with respect to PVN location), and figure 7D summarizes the results in a bar graph. As shown here six animals showed significant ultrasound-induced neuromodulation in the PVN, while none of the control animals showed such changes (or U/S-induced modulation of LPS-induced hyperglycemia).
